# Proteomics analysis reveals the effect of 1α,25(OH)_2_VD_3_-glycosides on development of early testes in piglets

**DOI:** 10.1038/s41598-021-90676-8

**Published:** 2021-05-31

**Authors:** Haodong Chen, Kathrin Bühler, Yan Zhu, Xiongwei Nie, Wanghong Liu

**Affiliations:** 1 College of Animal Science and Technology, Huazhong Agricultural University, Hongshan District, No.1 Shizishan Road, Wuhan, 430070 China; 2 National Engineering and Technology Research Center for Livestock, Wuhan, 430070 China; 3 The Breeding Swine Quality Supervision and Testing Center, Ministry of Agriculture, Wuhan, 430070 China; 4 Herbonis Animal Health GmbH, Rheinstrasse 30, CH-4302 Augst BL, Switzerland

**Keywords:** Proteomics, Developmental biology

## Abstract

1α,25(OH)_2_VD_3_ is the most active form of VD3 in animals. It plays an important role in regulating mineral metabolism but also in reproduction. Testes are the main reproductive organs of male mammals. Our research aims to reveal the effect of 1α,25(OH)_2_VD_3_-glycosides on development of early testes in piglets. 140 weaned 21-day old piglets were selected. The piglets were randomly divided into four groups and were fed a commercial diet supplemented with 0, 1, 2 and 4 μg/kg of 1α,25(OH)_2_VD_3_, provided as 1α,25(OH)_2_VD_3_-glycosides. Sixty days after the start of the experiment, at piglet age 82 days, testes were harvested. The morphology and histology of early testicular development were assessed. In addition, the proteomic TMT/iTRAQ labelling technique was used to analyse the protein profile of the testes in each group. Western blotting was applied to verify the target of differentially abundant proteins (DAPs). The analysis of morphology and histology of testes showed that a certain concentration of 1α,25(OH)_2_VD_3_-glycosides had a positive and significant effect on testicular development. And the results of proteomics analysis showed that of the identified 132,715 peptides, 122,755 were unique peptides. 7852 proteins, of which 6573 proteins contain quantitative information. Screening for DAPs focused on proteins closely related to the regulation of testicular development such as steroid hormone synthesis, steroid biosynthesis, peroxisome and fatty acid metabolism pathways. These results indicated that 1α,25(OH)_2_VD_3_ is involved in the regulation of early testicular development in piglets. At the same time, these findings provide valuable information for the proteins involved in the regulation of testicular development, and help to better understand the mechanisms of 1α,25(OH)_2_VD_3_ in regulating the development of piglets’ testes.

## Introduction

Vitamin D3 (VD3) is a fat-soluble vitamin that is produced in the skin from 7-dehydrocholesterol. VD itself is not biologically active and needs two conversion steps to become the metabolic active form, 1α,25-dihydroxycholecalciferol (1α,25(OH)_2_VD_3_)^1^. 1α,25(OH)_2_VD_3_ plays an important role in the organism. In addition to the well-known influences on calcium and phosphorus metabolism^2^, 1α,25(OH)_2_VD_3_ has also been discovered to have an influence on the reproductive system such as the reproduction and testicular development of male animals^3,4^. Furthermore, the presence of the vitamin D receptor (VDR) in rodents' testes and seminiferous tubules has been described^5^. After intravenous injection of [3H] 25-hydroxycholecalciferol, Johnson et al. (1985) found that high concentrations of 1α,25(OH)_2_VD_3_ were expressed in the kidney, epididymis and testis^6^. Research data from Kidroni et al. (1983) suggest that 1α,25(OH)_2_VD_3_ plays a role in rat reproductive tissues^7^. In addition, VDR expression was found in the testes of other organisms such as roosters, rams and humans^8–10^. VDR and VD-related metabolic enzymes can be clearly expressed in human testis, the ejaculation tract and in mature sperm. These results indicate that VD is essential for sperm production and maturation of human sperm^11^. VDR can also mediate a non-genomic increase in intracellular calcium concentration, increasing sperm motility. Furthermore, VD signalling has a positive effect on semen quality^4^.

It is acknowledged that it is the biologically active form of VD3, 1α,25(OH)_2_VD_3_, and not VD3 itself, that actually plays a key role in male reproduction^1,12^. Nevertheless, in livestock and poultry farming, VD supplementation is usually achieved using VD itself as the use of synthetic 1α,25(OH)_2_VD_3_ in livestock nutrition is too costly. On the other hand, a few plants such as *Solanum glaucophyllum* naturally produce 1α,25(OH)_2_VD_3_ in a glycosidic form^13^. 1α,25(OH)_2_VD_3_-glycosides have been shown to have the same or even better effects than synthetic 1α,25(OH)_2_VD_3_^14^. However, there is only a limited amount of research on the influence of 1α,25(OH)_2_VD_3_-glycosides from plant origin on reproductive development.

In this study, we explored the effects of different doses of 1α,25(OH)_2_VD_3_, provided as 1α,25(OH)_2_VD_3_-glycosides from *Solanum glaucophyllum* on early testicular development in piglets. Through proteomics analysis of testicular tissues from 82-day-old piglets, we revealed the regulatory mechanisms of the cumulative effect of 1α,25(OH)_2_VD_3_ on early testis development. Our experiment aims to provide a reference for future research on improving the life-long spermatogenic ability and fertility of boars.

## Materials and methods

### Feed and animal grouping

A total of 140 healthy 21-day-old weaned male piglets (Duroc x Landrace x Yorkshire) with a body weight range of 6.8 – 7.0 kg were selected for this experiment. The piglets were randomly divided into 4 groups of 35 animal each. The animals received an early nursery diet from 21 – 42 days of age and a late nursery diet from 43 – 81 days of age. Composition and nutritional values of the diets are given in Table 1. Basal VD supplementation was 1000 IU/kg_._ The nursery diets (S1–S4) were supplemented with 1α,25(OH)_2_VD_3_-glycosides by adding 0, 100, 200 or 400 g/t of a standardized complementary feed containing *Solanum glaucophyllum* (Herbonis Animal Health GmbH, Switzerland). The supplement provided 10 μg of 1α,25(OH)_2_VD_3-_glycosides (measured as free 1α,25(OH)_2_VD_3_)/kg. This resulted in an addition of 0, 1, 2, and 4 μg 1α,25(OH)_2_VD_3_-glycosides/kg diet (Table 2). Feed and water were provided ad libitum. Feed intake in the four treatment groups was similar.

**Table 1:** Basic diet nutrition formula (left) and analyzed nutrient content (right).

Raw material	Content %			Analyzed content	
	Early nursery	Late nursery		Early nursery	Late nursery
Corn grain(1st-grade)	22.2	32.8	DE kcal/kg	3323	3330
Extruded corn	10.0	8.0	CP %	18.4	17.7
Broken rice	22.0	15.0	EE %	40	44
Wheat bran	0.0	4.0	CF %	5.2	5.3
Steam fish meal	2.0	2.0	Moisture %	10.3	11.2
Soy protein concentrate	4.2	3.0	Ca %	0.83	0.78
Soybean meal (43%)	0.0	4.0	P %	0.58	0.57
Extruded soybean	12.0	12.0	Cu mg/kg	37	21
Fermented feed	2.0	2.0	Fe mg/kg	330	360
Dried porcine solubles	1.5	1.0	Zn mg/kg	1700	470
Whey protein concentrate	3.0	0	Mn mg/kg	81	96
Broken yeast extract	2.0	1.0	Lys %	1.42	1.27
Whey protein powder	11.0	6.0	Met % + Cys %	1.01	0.95
Soybean oil/ Palm oil (1:1)	0.0	1.0	Thr %	1.54	1.50
NaCl (99%)	0.1	0.2	Vitamin D_3_ (IU/kg)	990	987
Glucose	4.0	2.0			
Sucrose powder	0.0	2.0			
Premix in early nursery (4%)	4.0	0			
Premix in late nursery ( 4%)	0.0	4.0			
Total	100.0	100.0			

Premix in early nursery (4%): The premix provided per kg of diet 100,000 IU of vitamin A, 108 mg of vitamin B_1_, 260 mg of vitamin B_2_, 100 mg of vitamin B_6_, 0.8 mg of vitamin B_12_, 25,000 IU of vitamin D_3_, 1600 IU of vitamin E, 100 mg of vitamin K_3_, 100 mg of niacin, 55 mg of folic acid, 50 mg of pantothenic acid, 10 mg of biotin, 2250 mg of Fe, 1750 mg of Cu, 19,500 mg of Zn, 250 mg of Mn, 15 mg of I, 5 mg of Se. 10% Lys, 5% Met, 13.2% of Ca, 4.7% of P, 2.5% of NaCl.

Premix in late nursery (4%): The premix provided per kg of diet 75,000 IU of vitamin A, 80 mg of vitamin B_1_, 200 mg of vitamin B_2_, 75 mg of vitamin B_6_, 0.6 mg of vitamin B_12_, 25,000 IU of vitamin D_3_, 1200 IU of vitamin E, 75 mg of vitamin K_3_, 75 mg of niacin, 45 mg of folic acid, 40 mg of pantothenic acid, 8 mg of biotin, 3500 mg of Fe, 2250 mg of Cu, 1500 mg of Zn, 250 mg of Mn, 15 mg of I, 7 mg of Se. 12% Lys, 5% Met, 13.5% of Ca, 4.6% of P, 2.5% of NaCl.

**Table 2:** Experimental treatments.

Treatment	Nr of Piglets	VD_3_(IU/kg)	1α,25(OH)_2_VD_3_-glycosides^1^ (μg/kg)
S1	35	1000	0
S2	35	1000	1
S3	35	1000	2
S4	35	1000	4

^**1**^1α,25(OH)_2_VD_3_ was provided as 1α,25(OH)_2_VD_3_-glycosides from plant origin.

At the end of the trial at 82 days of age, both testes from each piglet were surgically removed, marked, weighed with an electronic balance and the volume of both testes was measured. The collected fresh right testis from each piglet was frozen immediately in liquid nitrogen and stored at − 80℃ until further analysis. The left testis was fixed in 4% pH 7.4 paraformaldehyde for subsequent testicular tissue sections measurements.

### Testicular phenotype data statistics

The volume of both testes was measured using the drainage method. Water was added to a measuring cylinder and the volume V_1_ was recorded. The entire testis was then completely immersed into the measuring cylinder and the volume V_2_ was recorded. The volume of the testis was calculated as: V_t_ = V_2_—V_1_. The testes on both sides of each group of piglets were weighed and volumetrically measured.

**Figure 1:**
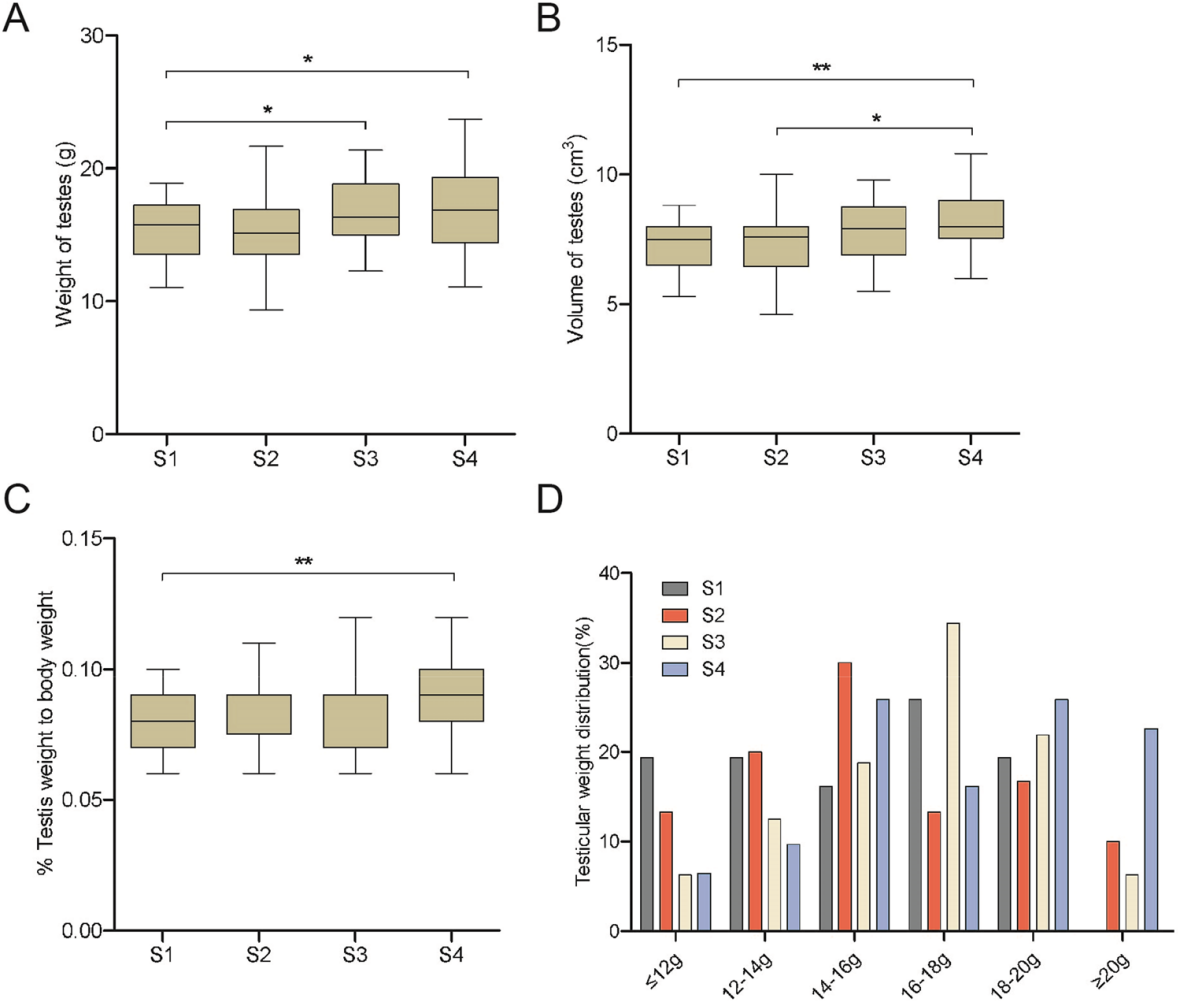
Data analysis of testicular development phenotype traits of piglets. (**A**) Testicular weight in g at 82 days of age. (**B**) Testicular volume in cm^3^ at 82 days of age. (**C**) Testis to body weight ratio at 82 days of age. (**D**) Testicular weight distribution for each treatment. The values shown are the means ± SEM (Significant difference: *P < 0.05; **P < 0.01; ***P < 0.001). S1: control diet; S2: control diet supplemented with 1 μg/kg 1α,25(OH)_2_VD_3_; S3: control diet supplemented with 2 μg/kg 1α,25(OH)_2_VD_3_; S4: control diet supplemented with 4 μg/kg 1α,25(OH)_2_VD_3._ 1α,25(OH)_2_VD_3_ was provided as 1α,25(OH)_2_VD_3_-glycosides from plant origin (n = 31, 30, 32, 31 for S1, S2, S3 and S4, respectively).

In order to describe the weight gain of piglets in each group more accurately, we introduced a testes to body weight ratio index: This index is calculated as the ratio of the weight of the testes collected from each piglet to the weight of the piglet at the end of the trial. This calculation reduces the error caused by the difference among the weight of the piglets. In order to better understand the testicular weight gain of each group, the weight distribution of the testes in each group was analysed (Fig. 1).

### HE staining of testicular tissue sections

**Figure 2:**
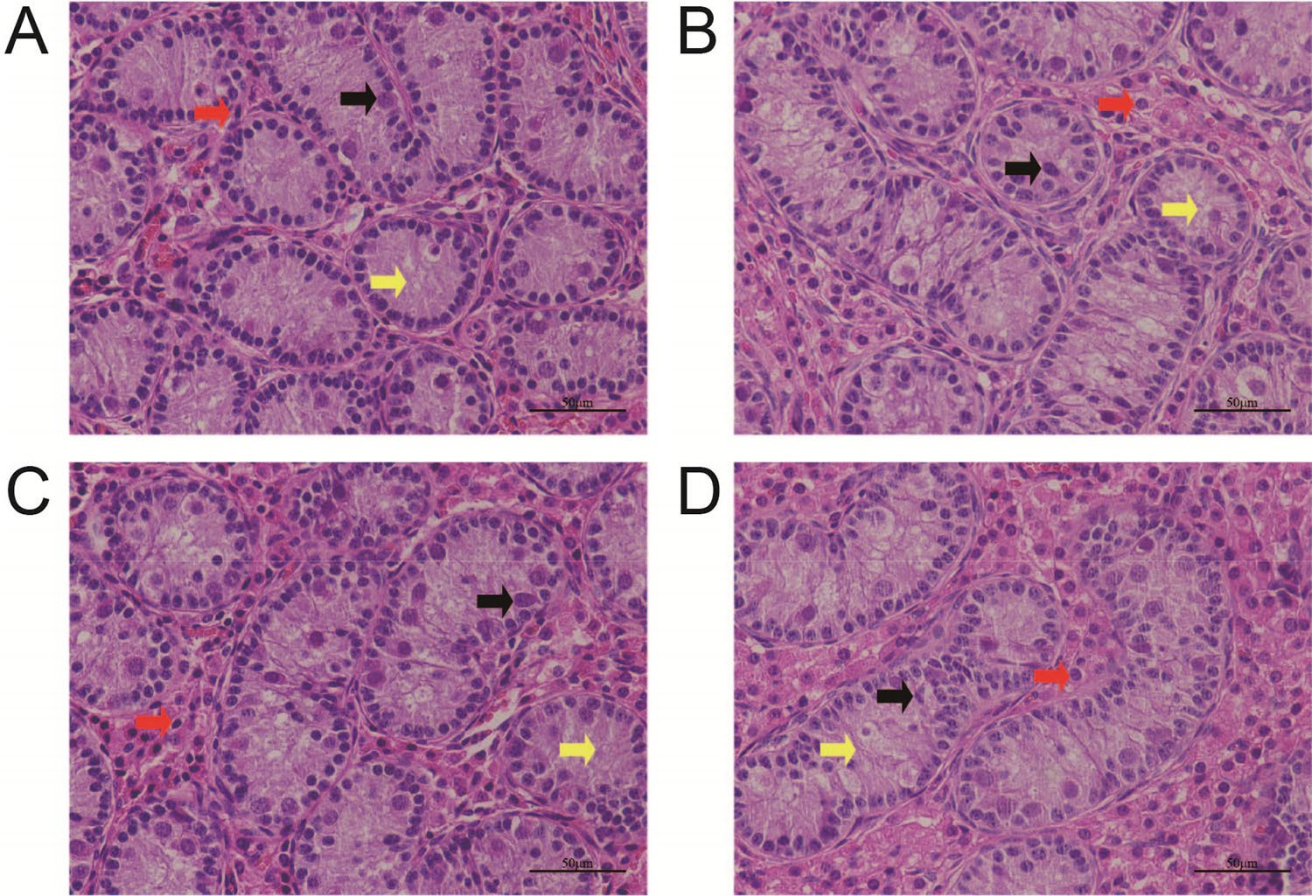
Paraffin section of testes at 82 days of age (HE staining). Black arrows indicate Sertoli cells (SC), red arrows Leydig cells (LC) and yellow arrows seminiferous tubules (ST). scale is 50 μm. (**A**) control diet; (**B**) control diet supplemented with 1 μg/kg 1α,25(OH)_2_VD_3_; (**C**) control diet supplemented with 2 μg/kg 1α,25(OH)_2_VD_3_; (**D**) control diet supplemented with 4 μg/kg 1α,25(OH)_2_VD_3_. 1α,25(OH)_2_VD_3_ was provided as 1α,25(OH)2VD3-glycosides from plant origin.

Five piglets were randomly selected from each group and the left testis was used for paraffin section and HE staining (Fig. 2). To obtain the tissue, the middle position of the testis was selected for crosscutting. The tissue was immersed in 4% paraformaldehyde for 24 h and then transferred to processing cassettes, dehydrated through a serial alcohol gradient, and embedded in paraffin wax blocks. Before staining, 5-um-thick testicular tissue sections were dewaxed in xylene, rehydrated through decreasing concentrations of ethanol, and washed in PBS. Staining was done with haematoxylin and eosin (H&E). After staining, sections were dehydrated through increasing concentrations of ethanol and xylene. In each group, 5 HE-stained sections were randomly selected and the morphological characteristics of testicular seminiferous tubules were observed under 40 × 10 magnification. Four fields of vision were randomly selected in each section. The area of near-round seminiferous tubules was measured with Scion Image software. In addition, the number of Leydig cells and Sertoli cells was counted in the same four fields of vision.

### Protein extraction

The whole testis was cut into small pieces with surgical scissors, and the samples were grinded by liquid nitrogen into a cell powder and then transferred to a 5-mL centrifuge tube. After that, four volumes of lysis buffer (8 M urea, 1% Protease Inhibitor Cocktail) were added to the cell powder, followed by sonication three times on ice using a high intensity ultrasonic processor (Scientz). The remaining debris was removed by centrifugation at 12,000 g at 4 ℃ for 10 min. Finally, the supernatant was collected, and the protein concentration was determined with BCA kit according to the manufacturer’s instructions^15^.

### Trypsin digestion

For digestion, the extracted testicular protein solution was reduced with 5 mM dithiothreitol for 30 min at 56 ℃ and alkylated with 11 mM iodoacetamide for 15 min at room temperature in darkness. The protein sample was then diluted by adding 100 mM TEAB to urea concentration less than 2 M. Finally, trypsin was added at 1:50 trypsin-to-protein mass ratio for the first digestion overnight and was added at 1:100 trypsin-to-protein mass ratio for a second 4 h-digestion immediately after the overnight digestion^16^.

### TMT labelling

After trypsin digestion, peptide was desalted by Strata X C18 SPE column (Phenomenex) and vacuum dried. Peptide was reconstituted in 0.5 M TEAB and processed according to the manufacturer’s protocol for TMT kit (Thermo Fisher Scientific). Briefly, one unit of TMT reagent were thawed and reconstituted in acetonitrile. The peptide mixtures were then incubated for 2 h at room temperature and pooled, desalted and dried by vacuum centrifugation. The samples of the four treatments S1, S2, S3, and S4 were labelled with TMT isobaric labels 126, 127, 128, and 129, respectively. The TMT labelling procedure was carried out three times for the biological replicates^17^.

### HPLC fractionation

The tryptic peptides were fractionated by high pH reverse-phase HPLC using Agilent 300Extend C18 column (5 μm particles, 4.6 mm ID, 250 mm length). In short, peptides were first separated with a gradient of 8% to 32% acetonitrile (pH 9.0) over 60 min into 60 fractions. Then, the peptides were combined to 18 fractions and dried by vacuum centrifuging^18^.

### LC–MS/MS analysis

The tryptic peptides were dissolved in 0.1% formic acid (solvent A), directly loaded onto a home-made reversed-phase analytical column (15-cm length, 75 μm i.d.). The gradient was comprised of an increase from 6 to 23% solvent B (0.1% formic acid in 98% acetonitrile) over 26 min, 23% to 35% in 8 min and climbing to 80% in 3 min then holding at 80% for the last 3 min, all at a constant flow rate of 400 nL/min on an EASY-nLC 1000 UPLC system.

The peptides were subjected to NSI source followed by tandem mass spectrometry (MS/MS) in Q ExactiveTM Plus (Thermo) coupled online to the UPLC. The electrospray voltage applied was 2.0 kV. The m/z scan range was 350 to 1800 for full scan, and intact peptides were detected in the Orbitrap at a resolution of 70,000. Peptides were then selected for MS/MS using NCE setting as 28 and the fragments were detected in the Orbitrap at a resolution of 17,500. A data-dependent procedure that alternated between one MS scan followed by 20 MS/MS scans with 15.0 s dynamic exclusion. Automatic gain control (AGC) was set at 5E4. Fixed first mass was set as 100 m/z^19,20^.

### Database search

The resulting MS/MS data were processed using Maxquant search engine (v.1.5.2.8). Tandem mass spectra were searched against Proteome *Sus scrofa* database (https://www.uniprot.org/taxonomy/9823) linked with a reverse decoy database. Trypsin/P was specified as cleavage enzyme allowing up to 2 missing cleavages. The mass tolerance for precursor ions was set as 20 ppm in First search and 5 ppm in Main search, and the mass tolerance for fragment ions was set as 0.02 Da. Carbamidomethyl on Cys was specified as fixed modification and oxidation on Met was specified as variable modifications. The FDR of PSM and protein was set to < 1%, the p-value was < 0.05, and minimum score for peptides was set > 40^21^.

### Bioinformatics methods

Gene Ontology (GO) annotation analysis of the differentially abundant proteins was derived from the UniProt-GOA database (www. http://www.ebi.ac.uk/GOA/). Proteins were classified by GO annotation based on three categories: biological process (BP), cellular component (CC) and molecular function (MF). We performed statistics on the distribution of DAPs in GO secondary annotations of each group. The Kyoto Encyclopaedia of Genes and Genomes (KEGG) database (http://www.genome.jp/kegg/) was used to annotate protein pathways. All DAPs database accessions or sequences were searched against the STRING database (version 10.5) for protein–protein interactions (PPI). Only interactions between the proteins belonging to the searched data set were selected, thereby excluding external candidates. STRING defines a metric known as “confidence score” to define interaction confidence. In this study, we used all interactions with a confidence score ≥ 0.7 (high confidence). Interaction network form STRING was visualized in R package “networkD3”. We visualized the interaction networks of DAPs in each group. In order to clearly show the protein–protein interaction (PPI), we selected the top 50 proteins with the closest interaction and plotted the PPI network of the three comparison groups S2/S1, S3/S1, and S4/S1^22^.

### Validation of the DAPs by Western blotting

The collected right testes from each piglet were cut into thin fragments and added to the lytic solution (protease and phosphatase inhibitors added to the lytic solution) at a ratio of 200 μL solution per 20 mg of tissue. The mixture was then homogenized until destruction. These samples were centrifuged at 4 ℃,12,000 g for 15 min, the supernatant was taken, and the protein was quantified and stored in − 80 ℃ refrigerator.

The extracted protein was electrophoresed on SDS-PAGE (Bio-Rad), and transferred to a PVDF membrane. After blocking with 5% skimmed milk powder for 2 h at room temperature, the diluted primary antibody was added and incubated at 4 °C overnight. The PVDF membrane was washed three times with TBST, and the HRP-labelled secondary antibody was diluted with TBST at a ratio of 1:10,000 and incubated for 2 h at room temperature with a shaker (Table 3). Proteins were detected using ECL chemiluminescence. The grey levels of related bands were read by TANON GIS software, and statistical analysis of triplicates in western blotting was done.

**Table 3:** Antibody information for Western blotting verification.

Antibody name	Antibody species	Brand	Art.No	Dilution ratio	Protein size
CYP11A1	Rabbit	Proteintech	13,363–1-AP	1:2000	50
HSD11B2	Rabbit	Proteintech	13,363–1-AP	1:1000	40
HSD17B4	Rabbit	Bioswamp	PAB41944	1:1000	88
PHYH	Rabbit	Proteintech	13,363–1-AP	1:1000	70
CAT	Rabbit	Bioswamp	PAB30818	1:2000	59
CYP19A1	Rabbit	Bioswamp	PAB31185	1:1000	48
FDFT1	Rabbit	Bioswamp	PAB34422	1:1000	48
PEX10	Rabbit	Bioswamp	PAB4433	1:1000	37
DHRS4	Rabbit	Bioswamp	PAB30397	1:1000	30
INSL3	Rabbit	Bioswamp	PAB34204	1:1000	15
β-Actin	Rabbit	Bioswamp	PAB36265	1:1000	42
Goat anti-Rabbit IgG	Goat	Bioswamp	SAB43714	1:10,000/1:20,000	/

### Statistical analysis

In this experiment, the data from phenotype traits of testicular development were assessed by Student's t-test using the standard statistics software SPSS (v.22.0, Chicago, IL, USA). Differences were considered to be significant if *P* < 0.05.

For statistical analysis of proteomics data, the average value of the three whole protein quantitative repeat experiments per treatment were used and then compared in pairs between control and treatment groups. The ratio is used as the final differential expression amount. The relative quantitative value of each group was converted to log2 to make the data conform to normal distribution. The two-sample two-tailed T test was used for statistical analysis. In case of *P* < 0.05, the change in the differential expression level exceeded 1.2 as the threshold for significant up-regulation and was smaller than 1/1.2 as the threshold for significant down-regulation.

### Ethics approval consent to participate

The animal protocols were approved by the Institutional Animal Care and Use Committee of Huazhong Agricultural University, China. The animal protocols and experiments were performed in compliance with the ARRIVE guidelines and the guidance of the Animal Ethics Procedures and Guidelines of the People's Republic of China.

### Consent for publication

All authors read and approved the final manuscript.

## Results

### Testicular development phenotype traits

In terms of testicular weight, S2 was similar to the control group (S1) (*P* > 0.05), whereas testicular weight of group S3 and S4 were significantly different (*P* < 0.05). A similar trend was found for testicular volume (S1 vs S2 and S3: *P* > 0.05; S1 vs S4: *P* < 0.01) (Fig. 1A,B). Testicular volume of S4 was with 8.46 cm^3^ significantly higher than in S2 with 7.59 cm^3^ (*P* < 0.05). The difference of the testis weight to body weight ratio index between animals fed treatment S2 and S3 was not significant compared to the control group (*P* > 0.05), In contrast, the difference between S4 and control group was significant (*P* < 0.01) with the ratio in treatment S4 being 12.75% higher than in S1 (Fig. 1C). Weight distribution showed that there were no testes ≥ 20 g in treatment S1. Testicular weight distribution was shifted to the right in accordance to higher levels of 1α,25(OH)_2_VD_3_-glycosides added to the diet (Fig. 1D).

### Morphological analysis of testicular development

**Figure 3:**
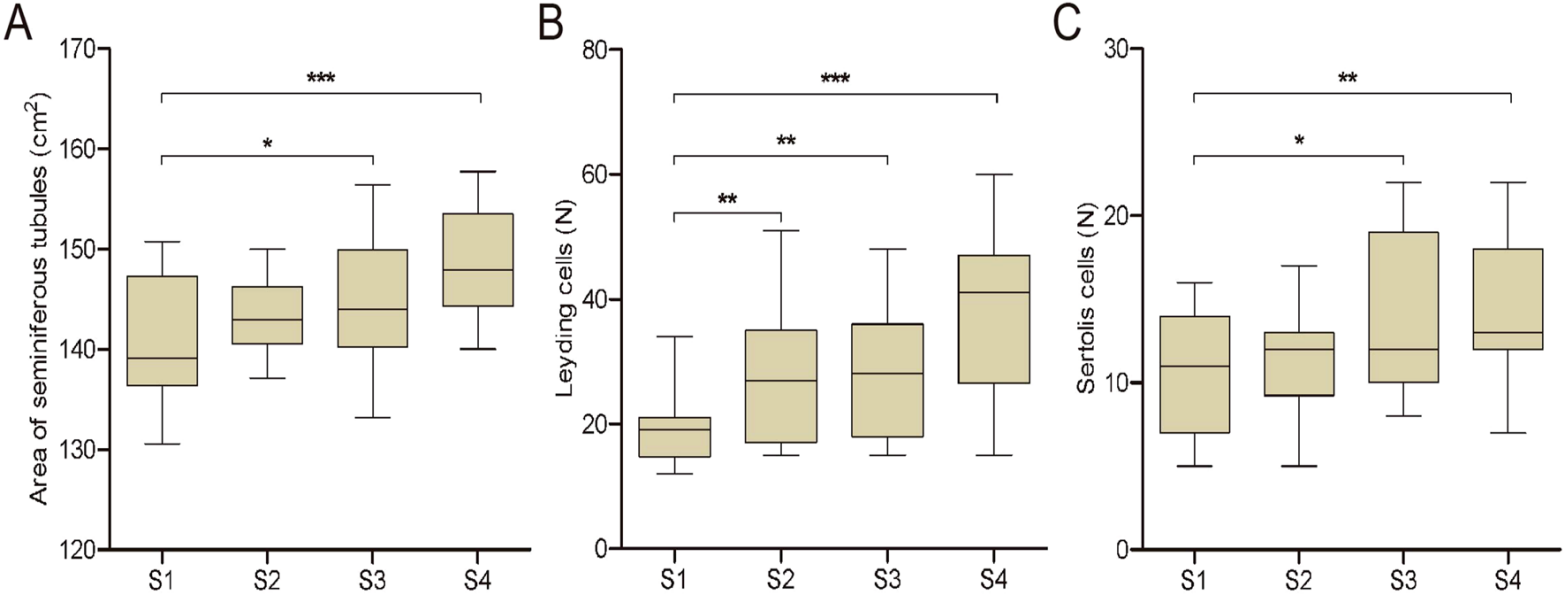
Morphological differences in testes of piglets at 82 days of age. (**A**) Area of seminiferous tubules. (**B**) Number of Leydig cells. C. Number of Sertoli cells. The values shown are the means ± SEM (Significant difference: *P < 0.05; **P < 0.01; ***P < 0.001). S1: control diet; S2: control diet supplemented with 1 μg/kg 1α,25(OH)_2_VD_3_; S3: control diet supplemented with 2 μg/kg 1α,25(OH)_2_VD_3_; S4: control diet supplemented with 4 μg/kg 1α,25(OH)_2_VD_3._ 1α,25(OH)_2_VD_3_ was provided as 1α,25(OH)_2_VD_3_-glycosides from plant origin (n = 20 per treatment).

The morphological analysis of the testes showed that the number of Leydig cells in animals from group S4, S3 and S2 were significantly higher than in the control group (*P* < 0.001 and *P* < 0.01, respectively), (Fig. 3B); Similarly, the number of Sertoli cells in S4 and S3 increased compared to the control group (*P* < 0.01 and *P* < 0.05, respectively) (Fig. 3C) as did the area of seminiferous tubules (*P* < 0.001 and *P* < 0.05, respectively) (Fig. 3A).

### Identification of peptides

A total of 815,971 secondary spectra were obtained by TMT quantitative proteomic analysis. After searching the library of protein theoretical data, the secondary spectrum of mass spectrometry obtained the available effective spectrum number of 248,564, and the spectrum utilization rate was 30.5%. A total of 132,715 peptides were identified through spectrum analysis. 122,755 of the identified peptides were unique peptides. A total of 7852 proteins were identified, 6573 of which were quantifiable indicating that at least one comparison group had quantitative information (Supplementary material: Table S1).

### Protein differential expression analysis

**Figure 4:**
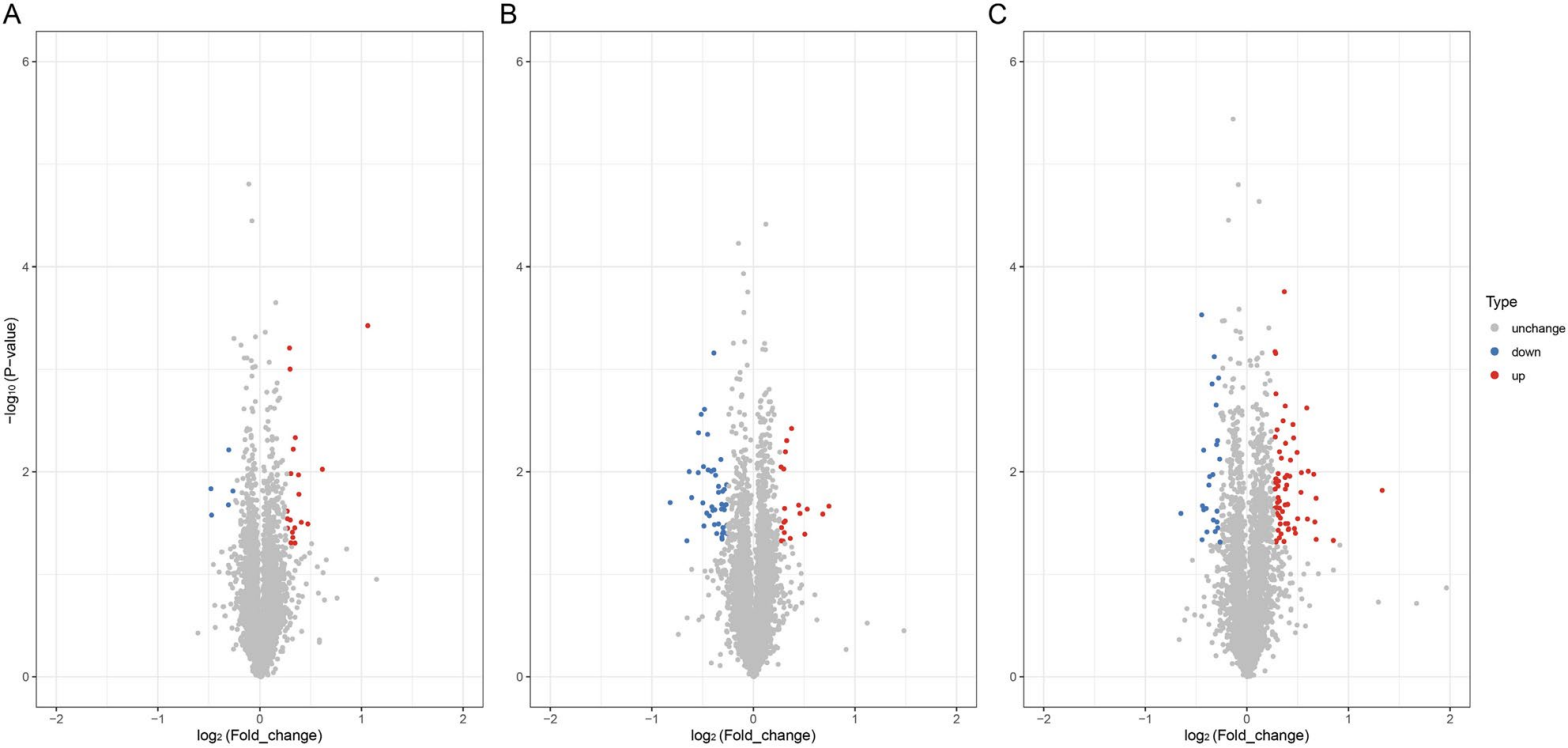
Volcano plot of differentially abundant proteins (DAPs) in each comparison group. (**A**) S2 vs S1; B. S3 vs S1; C. S4 vs S1. The horizontal coordinate represents the fold change of the differentially abundant proteins (log2). The vertical coordinate represents the logarithmic transformation value of the p-value. The red points indicate significantly up-regulated DAPs, the blue points indicate significantly down-regulated DAPs, and the grey points indicate proteins that were not differentially abundance. S1: control diet; S2: control diet supplemented with 1 μg/kg 1α,25(OH)_2_VD_3_; S3: control diet supplemented with 2 μg/kg 1α,25(OH)_2_VD_3_; S4: control diet supplemented with 4 μg/kg 1α,25(OH)_2_VD_3_. 1α,25(OH)_2_VD_3_ was provided as 1α,25(OH)_2_VD_3_-glycosides from plant origin.

Summary data of all DAPs in this study are shown in Table 4 (Supplementary material: Table S2). In addition, analysis of the volcano map revealed the identified DAPs (Fig. 4). It can be seen that DAPs in S3 and S4 were more significant and richer compared to the control group (S1). This is especially true for the comparison S4 vs S1, where the difference in protein expression levels between the two groups was the largest, which indicates that DAPs obtained by screening are more reliable.

**Table 4:** Summary of all differentially abundant proteins.

Comparison group	Up (> 1.2)	Down (< 1/1.2)
S2/S1	20	5
S3/S1	19	43
S4/S1	66	24

S1: control diet; S2: control diet supplemented with 1 μg/kg 1α,25(OH)_2_VD_3_; S3: control diet supplemented with 2 μg/kg 1α,25(OH)_2_VD_3_; S4: control diet supplemented with 4 μg/kg 1α,25(OH)_2_VD_3._ 1α,25(OH)_2_VD_3_ was provided as 1α,25(OH)_2_VD_3_-glycosides from plant origin.

### GO functional classification of the DAPs

**Figure 5:**
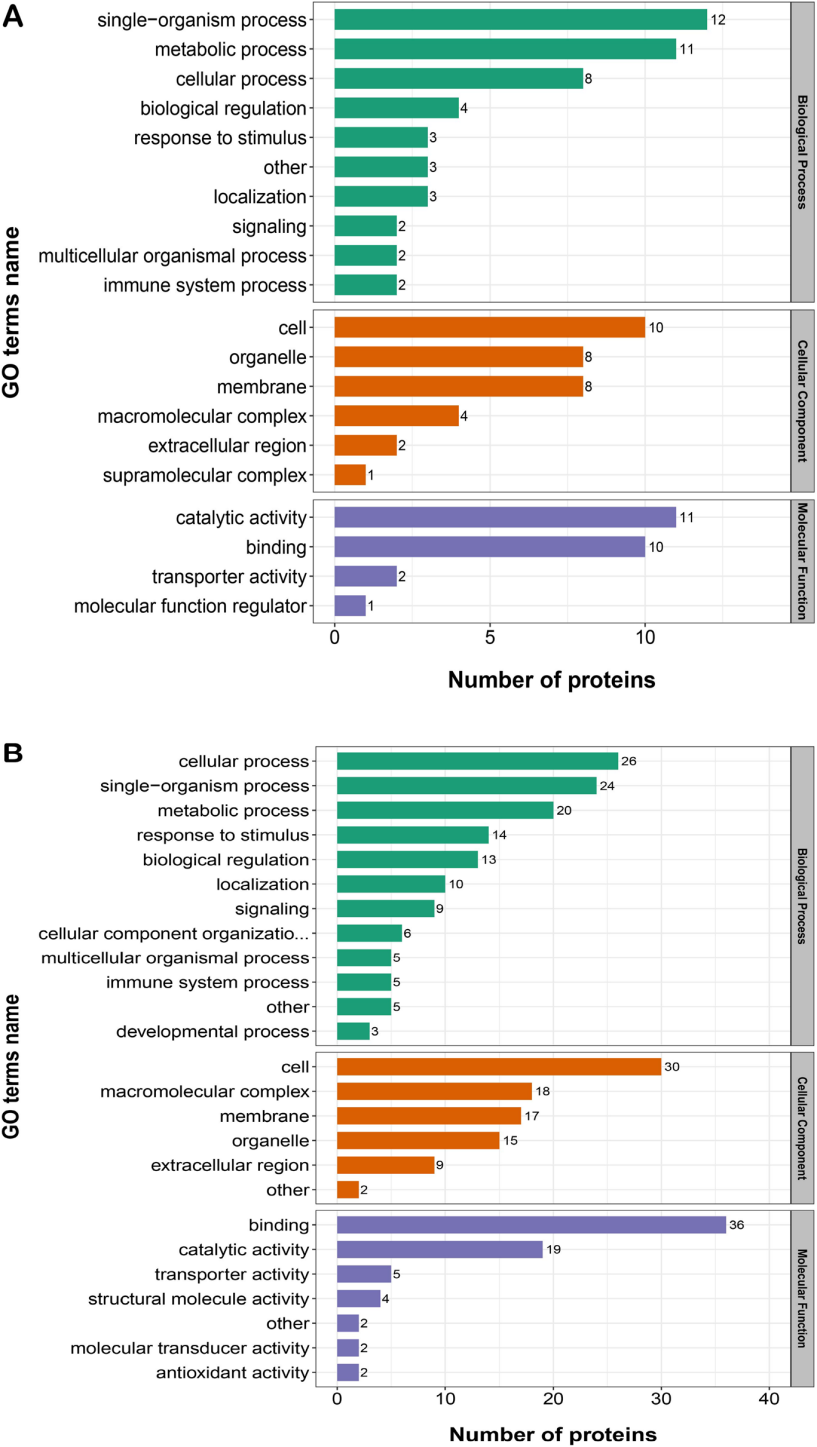

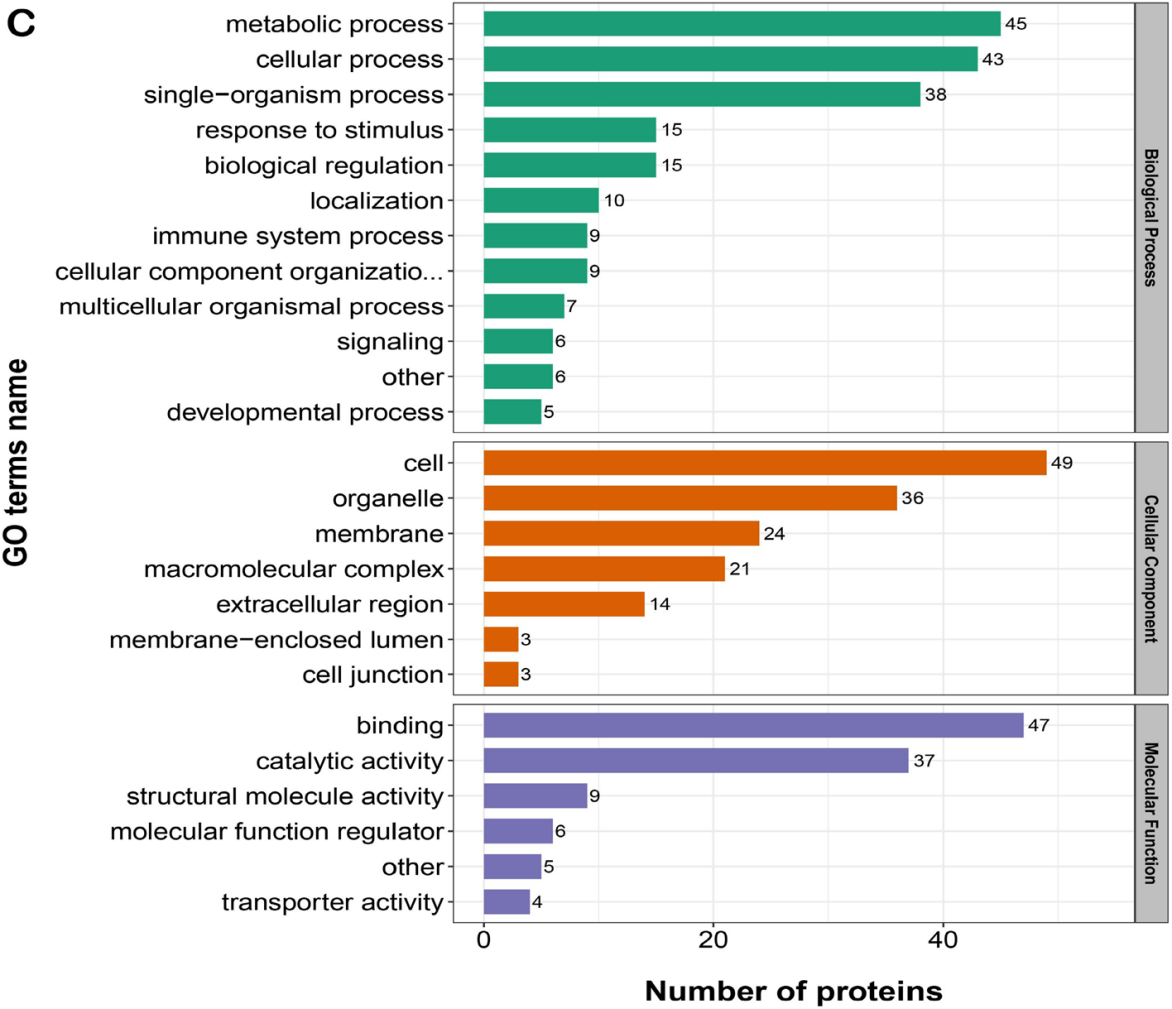
Statistical distribution chart of differentially abundant proteins (DAPs) under each Gene Ontology (GO) category in each comparison group. (**A**) S2 vs S1; (**B**) S3 vs S1; (**C**) S4 vs S1. The horizontal coordinate represents the number of proteins. The vertical coordinate represents the GO terms. S1: control diet; S2: control diet supplemented with 1 μg/kg 1α,25(OH)_2_VD_3_; S3: control diet supplemented with 2 μg/kg 1α,25(OH)_2_VD_3_; S4: control diet supplemented with 4 μg/kg 1α,25(OH)_2_VD_3._ 1α,25(OH)_2_VD_3_ was provided as 1α,25(OH)_2_VD_3_-glycosides from plant origin.

The GO enrichment analysis revealed that DAPs showed the enrichment trend of cellular process, single-organism process and metabolic process in terms of biological processes. The proportions in each comparison group (S2/S1, S3/S1, S4/S1) were: 62%, 50% and 60.6%, respectively. In the cellular component term, results showed the enrichment trend of cell, organelle, membrane and macromolecular complexes. In each comparison group, this category accounted for: 90.9%, 87.9% and 86.7%, respectively. In the molecular function term, GO enrichment analysis indicated the enrichment trend of catalytic activity and binding. The proportions in each comparison group were: 87.5%, 78.6%, and 77.8%, respectively (Fig. 5A–C). (Supplementary material: Table S3, Table S4, Table S5, and Table S6).

### Enrichment analysis of KEGG pathway of DAPs

**Figure 6:**
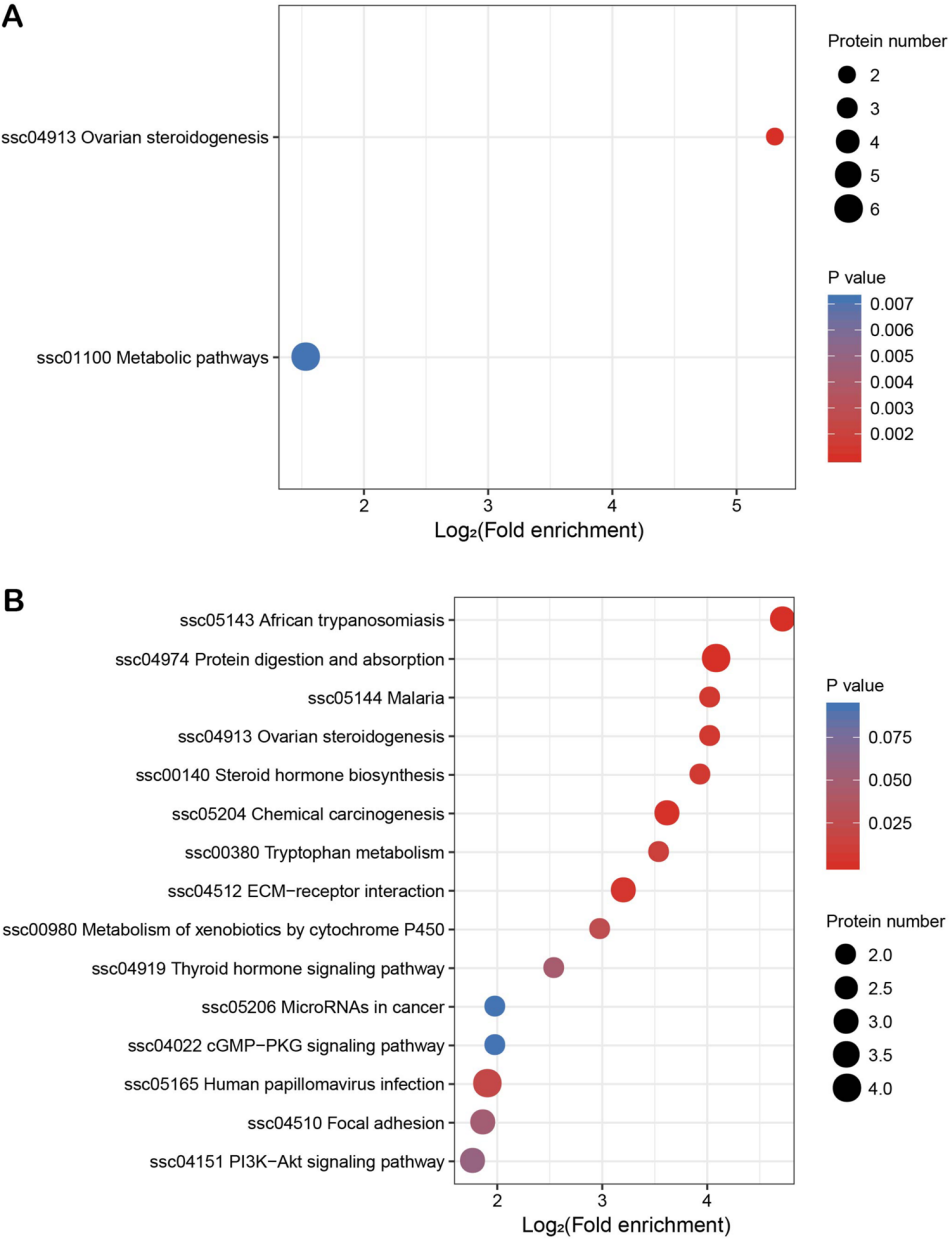

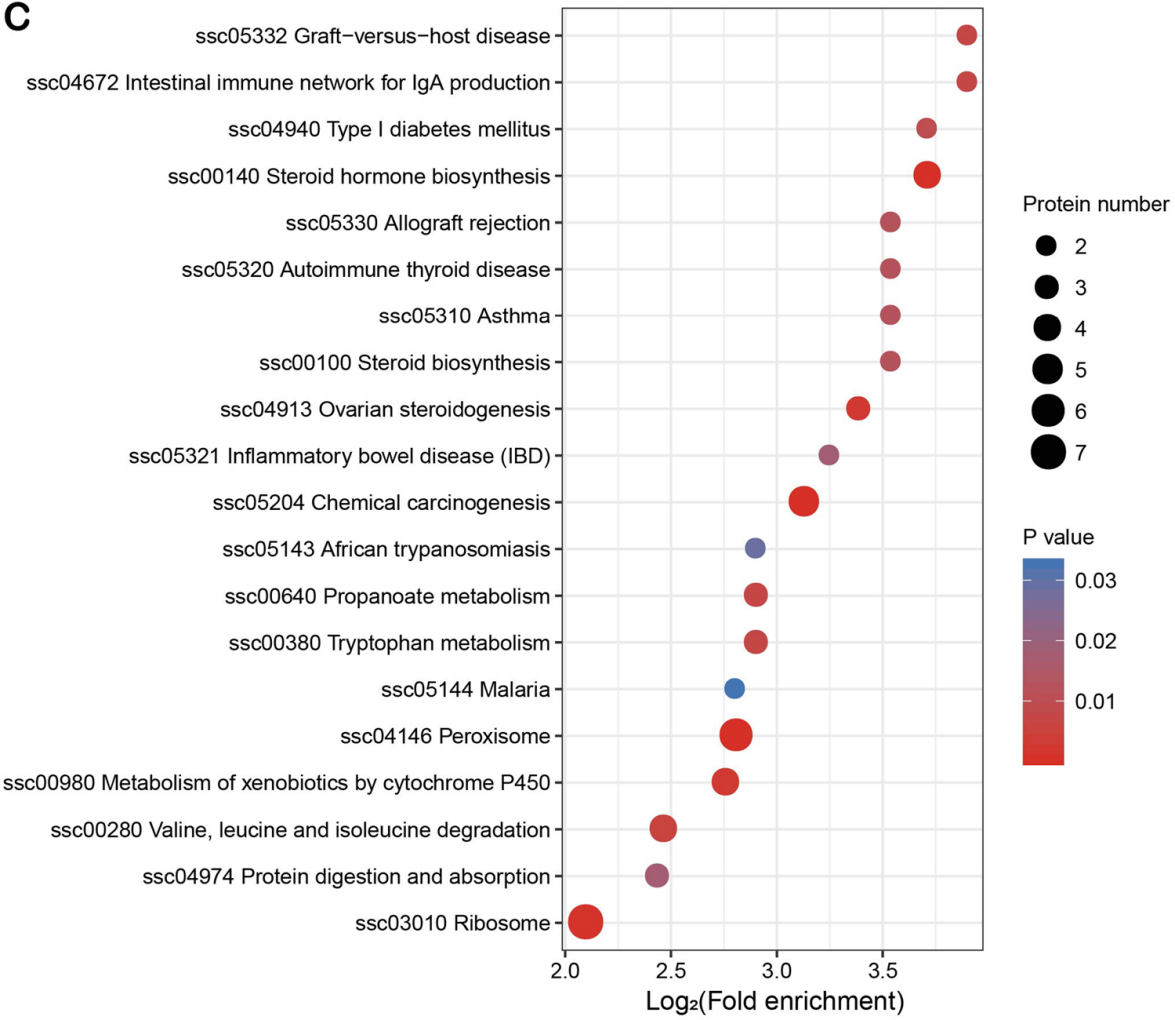
KEGG pathway enrichment bubble plot of differentially abundant proteins (DAPs). (**A**) S2 vs S1; (**B**) S3 vs S1; (**C**) S4 vs S1. The bubble chart gives the results of the top 20 classifications with the most significant enrichment. The vertical axis is the functional classification or pathway, and the horizontal axis is a log2 converted value of the proportion of differential proteins in this functional type compared to the multiple of the proportion of identified proteins. The circle color indicates enrichment significance (p-value), circle size indicates the number of differential proteins in functional classes or pathways. S1: control diet; S2: control diet supplemented with 1 μg/kg 1α,25(OH)_2_VD_3_; S3: control diet supplemented with 2 μg/kg 1α,25(OH)_2_VD_3_; S4: control diet supplemented with 4 μg/kg 1α,25(OH)_2_VD_3._ 1α,25(OH)_2_VD_3_ was provided as 1α,25(OH)_2_VD_3_-glycosides from plant origin.

KEGG pathway enrichment analysis showed that DAPs between experimental groups and the control group belonged to 28 pathways. This was based on the KEGG database (www.kegg.jp/kegg/kegg1.html), of which 8 pathways appeared in 2 or 3 comparison groups (Fig. 6A–C). These pathways are mainly focused on: Ovarian steroidogenesis, Steroid hormone biosynthesis, Protein digestion and absorption, Metabolism of xenobiotics by cytochrome P450, Tryptophan metabolism, Malaria, African trypanosomiasis, Chemical carcinogenesis, Peroxisome, Steroid biosynthesis, PI3K-Akt signalling pathway. Among them, steroid biosynthesis, steroid hormone synthesis and peroxisome pathways^23^ and DAPs such as CYP11A1^24^, CYP19A1^25,26^, FDFT1, PEX10^27^, CAT^28^, DHRS4^29^ are closely related to the regulation of testicular development and spermatogenesis. (Supplementary material: Table S7, S8, S9).

### Analysis of PPI network for the DAPs

**Figure 7:**
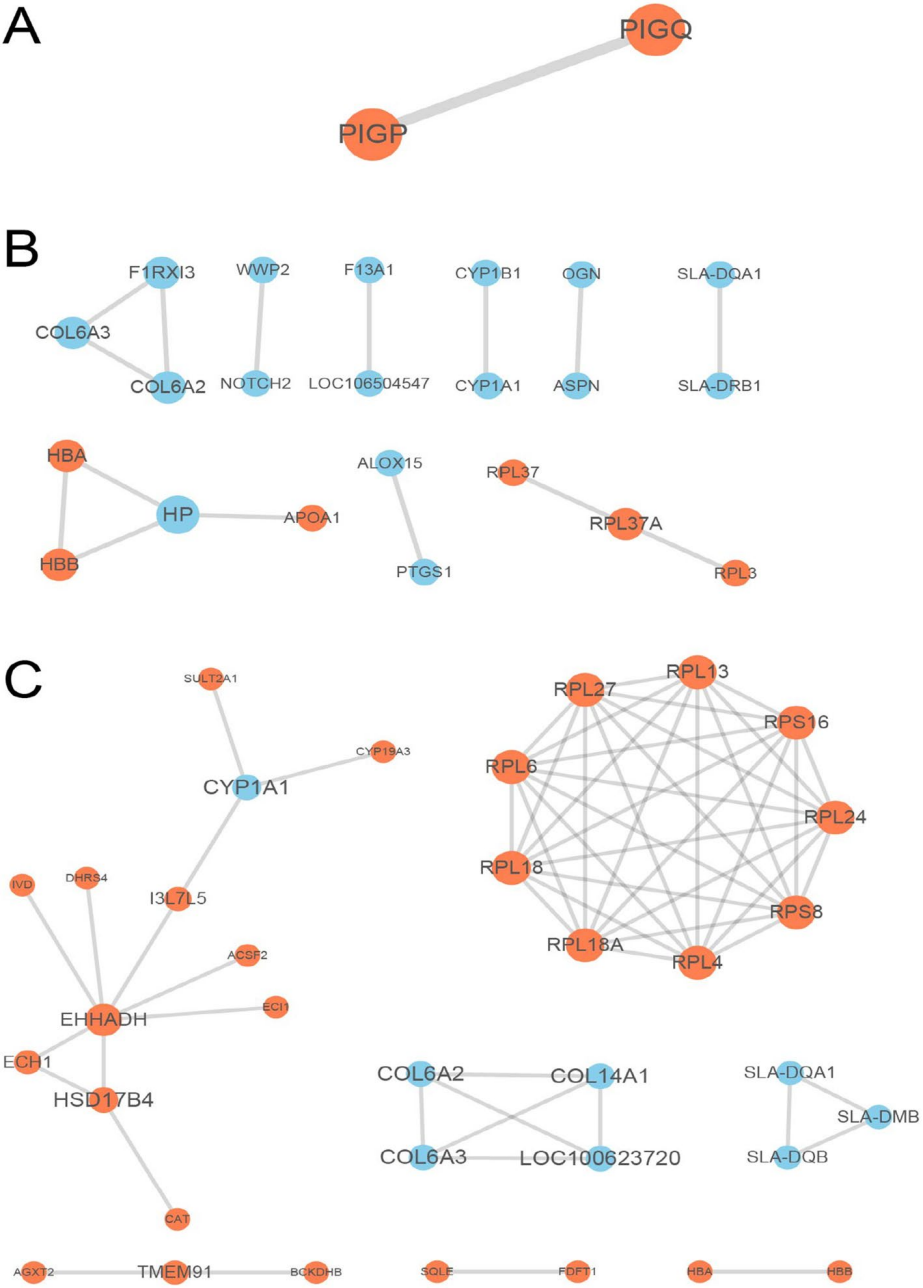
Protein–protein interaction (PPI) network generated for differentially abundant proteins (DAPs) using STRING. (**A**) S2 vs S1; (**B**) S3 vs S1; (**C**) S4 vs S1. We screened out the top 50 most closely interacting proteins and obtained all interactions exhibiting confidence scores ≥ 0.7 (high confidence). The circles in the figure indicate DAPs, and the red colour indicates significantly up-regulated DAPs, the blue colour indicates significantly down-regulated DAPs. The size of the circle represents the number of differential proteins and their interacting proteins. S1: control diet; S2: control diet supplemented with 1 μg/kg 1α,25(OH)_2_VD_3_; S3: control diet supplemented with 2 μg/kg 1α,25(OH)_2_VD_3_; S4: control diet supplemented with 4 μg/kg 1α,25(OH)_2_VD_3._ 1α,25(OH)_2_VD_3_ was provided as 1α,25(OH)_2_VD_3_-glycosides from plant origin.

Comparing the testes of the treatment groups with the control group (**N1**, **N2**, **N3**; Fig. 7), we found proteins in the interaction centre that connect important pathways, such as EHHADH, HSD17B4, ECH1, BCKDHA, CYP1A1, etc. They correspond to one or more pathways of fatty acid metabolism, peroxisomes, fatty acid biosynthesis, amino acid metabolism, lipid synthesis and metabolism, and similar pathways. These pathways are mainly involved in metabolic process, oxidation–reduction process, cellular process and other biological processes, as well as molecular functions such as protein binding, receptor binding, catalytic activity, and oxidoreductase activity.

### Validation of the DAPs by Western blotting

**Figure 8:**
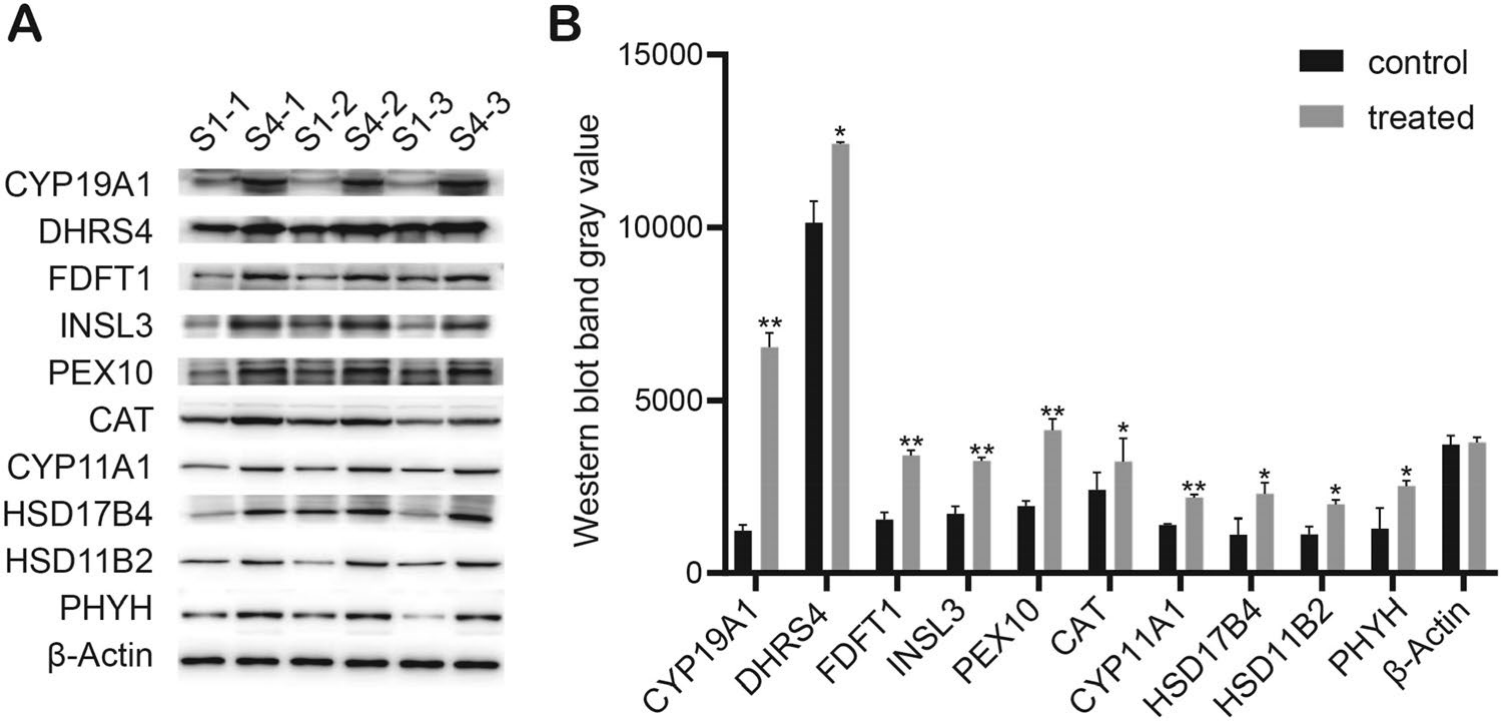
Validation of ten selected proteins in testicular tissue of animals from treatments S1 and S4 by Western blotting. (**A**) The abundance of CYP11A1, CYP19A1, HSD11B2, FDFT1, PEX10, PHYH, HSD17B4, CAT, DHRS4 and INSL3 proteins were analyzed by Western blotting in three replicates. The β-actin was used as an internal reference. (**B**) Comparison and verification of the quantitative results for selected proteins from the Western blotting. The values shown are the means ± SEM (Significant difference: *P < 0.05; **P < 0.01; ***P < 0.001). S1: control diet; S4: control diet supplemented with 4 μg/kg 1α,25(OH)_2_VD_3._ 1α,25(OH)_2_VD_3_ was provided as 1α,25(OH)_2_VD_3_-glycosides from plant origin (n = 3 per treatment).

In order to further verify the changes of the DAPs, we screened ten important functionally related proteins and verified their abundance in S4 and control group (S1) by Western blotting (Fig. 8). The verification results were consistent with the preliminary findings of TMT proteomics analysis. This indicated that the proteomics data were credible, and that some of the proteins such as CYP19A1, FDFT1, PEX10, CYP11A1, INSL3 requested further research.

## Discussion

VDR has been found to be present in various male animal reproductive tissues^4^, indicating the important role of 1α,25(OH)_2_VD_3_ in regulating male animal reproductive development. Testicular development and spermatogenic ability of breeding boars are key to improve productivity of the pig industry. This is also due to the fact that early testicular development of breeding boars is closely related to the lifelong spermatogenic ability^30–32^. Therefore, DAPs identified in this study may help to use TMT quantitative proteomics strategies to reveal the regulatory mechanism of early testicular development in boars.

Analysis of testicular development phenotype data in this study showed that 4 μg/kg of 1α,25(OH)_2_VD_3_-glycosides from *Solanum glaucophyllum* had a positive effect on the weight and volume of tested at 84 d of age. HE staining results furthermore indicated that the highest dosage of 1α,25(OH)_2_VD_3_ on top of 1000 IU VD promoted the proliferation of testicular cells and promoted the development of seminiferous tubules in piglets. This might eventually improve the spermatogenic ability of the adult boar.

The results of GO functional enrichment analysis revealed that these DAPs may be mainly enzymes with catalytic activity located in the organelles or cell membranes during cellular and metabolic processes.

It is obvious that the supplementation of 1α,25(OH)_2_VD_3_-glycosides, especially at the level of 4 μg/kg feed on top of a basal VD supplementation of 1000 IU/kg feed, improved testes growth indexes such as testicular weight, testicular volume and testes to body weight ratio compared to the control group. Studies with 25-hydroxy vitamin D-1α-hydroxylase-KO mice showed significantly lower testicular weight and testes to body weight ratio as well as reduced sperm count and motility compared with wild type mice^33^. It is known that testicular weight and testicular volume are positively correlated with testicular function such as the number of sperms produced by testes^34–36^. Similarly, the results of HE staining showed that the number of Sertoli cells and Leydig cells in the testes of piglets in trial group fed with higher dosage of 1a,25(OH)_2_VD_3_ increased significantly. An increase in the surface area of seminiferous tubules might improve spermatogenesis as the two parameters are closely related^37^. During the process of spermatogenesis, Sertoli cells provide a stable microenvironment, which is the basis for spermatogenesis before sexual maturity and maintenance of spermatogenesis after adulthood^38^. Furthermore, Leydig cells secrete testosterone and promote spermatogenesis and testicular development^39^. Finally, the growth of testes is mainly manifested in the significant increase in the diameter of seminiferous tubules^40^. The phenotypic changes observed in tested of piglets fed 4 μg/kg of 1a,25(OH)_2_VD_3_-glycosides from plant origin indicate that the early testicular development of piglets was positively influenced. The early development of piglets' testes is a complex process regulated by multiple pathways. The enrichment analysis of the KEGG pathway of DAPs done in this study gives indications that these pathways might be closely related to regulatory processes such as Steroid biosynthesis, Steroid hormone synthesis, Peroxisome, Fatty acid metabolism pathway and others.

The cholesterol side chain lyase (CYP11A1) is a key enzyme that regulates the synthesis of steroid hormones. It mainly catalyses the conversion of C27 cholesterol to the first C21 steroid pregnenolone. This is the first and rate-limiting step of steroid hormone synthesis^41^, as pregnenolone is converted into the progestin hormone progesterone by a bifunctional enzyme complex. Pregnenolone and progesterone are the starting materials for three groups of steroids: C21 steroids of glucocorticoids and mineralocorticoids, C19 steroids of androgens, and C18 steroids of oestrogens^42–44^. Compared with the control group, the expression of CYP11A1 in treatment S4 was significantly up-regulated. Cholesterol cleavage is the rate-limiting step in the synthesis process of testosterone (including oestrogen). Previous studies have shown that increased expression of CYP11A1 can promote testosterone secretion in testicular Leydig cells and maintain normal sexual function^24^.

CYP19A1 is a cytochrome P450 monooxygenase that catalyses the conversion of C19 androgen, androstene-4,17-dione (androstenedione) and testosterone to C18 oestrogen, estrone and oestradiol, respectively^45^. It is the rate-limiting enzyme of oestrogen biosynthesis and is mainly involved in ovarian steroidogenesis and steroid hormone synthesis. In this study, the expression of CYP19A1 in animals fed the highest amount of 1a,25(OH)_2_VD_3_-glycosides was significantly up-regulated (2.51-fold up-regulation) compared to the control group. Cytoplasmic co-expression of metabolic enzymes in VDR and Leydig cells suggests that VD affects male reproductive hormone production^46^. 1α,25(OH)_2_VD_3_ is able to stimulate the aromatase gene expression in purified immature rat Sertoli cells^47^. Oestrogen plays an important role in the absorption of water during the transport of sperm from the testes to the epididymis. Low levels of oestrogen can cause changes in osmotic pressure, leading to sperm dysfunction^48^. Oestrogen acts both as a sex steroid hormone and a growth or differentiation agent and can promote growth and differentiation of testicular cells. Sertoli cells and some germ cells in testes are also targets of oestrogen^25,49^. The role of aromatase/oestrogen is not only in the development process but also in maintaining spermatogenesis, and in the final maturation of sperm^26^. Therefore, oestrogen is necessary for normal male reproduction as it is a potential regulator of spermatogenesis in many species, including humans. Thus, it can be inferred that the increase of androgen gene expression and the increase of oestrogen gene expression in piglets of experimental group S4 has a beneficial effect on testicular development and spermatogenesis^50^. However, it is worthy of discussion and further research to determine which effect oestrogen levels in the testes of piglets will have on testicular development. In addition, the expression of HSD11B2 in S4 was significantly increased. It catalysed the glucocorticoid cortisol to the inactive metabolite cortisone, thereby preventing the activation of the mineralocorticoid receptor. In tissues not expressing mineralocorticoid receptors, such as testes, this can protect cells from growth inhibition and/or pro-apoptotic effects of cortisol^51^.

The expression levels of FDFT1, SQLE, and EBP in S4 increased significantly. These are the three key enzymes in the steroid biosynthesis pathway. The FDFT1 gene encodes a membrane-associated enzyme located at the branching point in the mevalonate pathway. The FDFT1 protein is the first specific enzyme in cholesterol biosynthesis and can catalyse the dimerization of two molecules of farnesyl diphosphate to form squalene in a two-step reaction. SQLE protein catalyses the stereospecific oxidation of squalene to (S)-2,3-epoxy squalene, and is thought to be one of the rate-limiting enzymes in this pathway. Finally, EBP catalyses the conversion of Delta(8)-sterols to their corresponding Delta(7)-isomers, and finally cholesterol through the action of 7-dehydrocholesterol reductase. The increased expression of FDFT1, SQLE and EBP is likely to result in a higher production of cholesterol, which can be used in steroid hormone synthesis and steroid degradation pathways. In summary, the findings discussed above indicate that in regard to the steroid hormone synthesis pathway, the testes of animals in group S4 showed improved development compared to the control treatment and thus a positive effect of 1a,25(OH)_2_VD_3_-glycosides.

Peroxisomes are essential organelles that are ubiquitous in eukaryotic cells. They are vesicles surrounded by a layer of unit membranes and they play a key role in redox signalling and maintaining lipid homeostasis^52,53^. In this study the proteomics analysis of treatments S4 and S1 showed that the peroxisomal biosynthetic pathway was significantly up-regulated in group S4, especially the enzymes PEX10, PHYH, HSD17B4, EHHADH, CAT, DHRS4.

The biogenesis of peroxisomes relies on a common class of evolutionary conserved genes referred to as “PEX genes”^54^. PEX10, PEX2 and PEX12 encode integral membrane proteins and constitute the Ring-finger complex which is required for receptor recycling. The disruption of the Drosophila PEX genes (PEX2 and PEX10) leads to spermatogenesis defects, including failure of spermatocyte growth and cytokinesis. Therefore, PEX, especially PEX2 and PEX10, are crucial for male Drosophila testicular development and reproduction^55^. Its orthologs are also present in mice and humans, although there is lack of research on the PEX gene in the field of mammalian reproduction and development^56–58^.

Peroxisomes are also involved in the metabolism of animal fatty acids, especially Very Long Chain Fatty Acids (VLCFA ). In most organs, the level of VLCFA is normally rather low. However, in some selected animal tissues such as retina, spermatozoa and the myelin sheath, the content of VLCFA is elevated^59^. Increased levels of VLCFA enhance defects in Drosophila spermatogenesis, while decreased levels of VLCFA alleviate these problems. Therefore, maintaining proper VLCFA level is essential for male spermatogenesis. PHYH, HSD17B4 and EHHADH participate in the fatty acid oxidation process and play an important role in it. PHYH converts phytanyl-CoA to 2-hydroxyphytanyl-CoA and participates in the alpha-oxidation of 3-methyl branched fatty acid of peroxisome. HSD17B4 catalyses the formation of 3-ketoacyl-CoA intermediates from both straight-chain and 2-methyl-branched-chain fatty acids. The N-terminal region of the protein encoded by EHHADH contains enoyl-CoA hydratase activity, while the C-terminal region contains 3-hydroxyacyl-CoA dehydrogenase activity, which can catalyse the peroxisomal fatty acid β-oxidation of long-chain fatty acids. Both HSD17B4 and EHHADH are bifunctional enzymes, which participate in the peroxisome β-oxidation pathway of fatty acids. A defect of peroxisome β-oxidation in Sertoli cells may be the cause of lipid accumulation. In mice, the gradual accumulation of lipids leads to complete atrophy of the seminiferous epithelium, steatosis of the testes, and retardation of testicular development^60^. Lipid metabolism thus plays a vital role in maintaining testicular growth and development^61^. The findings of Haiyang et al. (2010) also indicated that the PEX gene is essential for maintaining appropriate VLCFA levels^55^. During spermatogenesis the different steps are very sensitive to the level of oxidation inside and outside of the cell. The increased expression of CAT in testicular tissue can effectively remove excessive reactive oxygen species (ROS) in the oxidizing environment, thereby protecting the development and maturation of the testes and thus ensuring proper spermatogenesis^28^. Research by Borges et al. (2018) found that VD deficiency can lead to decreased lipid β oxidation in the liver of mice and which can eventually cause liver diseases such as obesity^62^. However, related studies on VD metabolism and β-oxidation in the field of reproductive development have not yet been published. Our research results showed that the addition of 4 μg/kg 1α,25(OH)_2_VD_3_ in the diet increased β-oxidation in the testicular tissue of piglets and to ultimately promote testicular development.

In addition, DHRS4, INSL3 and other related genes also play an important role in the development of testes and spermatogenesis in male animals^29,63–65^. Therefore, compared to the control group, the enzymes of PEX10, PHYH, HSD17B4, EHHADH, CAT, DHRS4 and INSL3 were significantly up-regulated in the peroxisome biosynthetic pathway, which also confirmed that the positive role of 1α,25(OH)_2_VD_3_ in regulating the testicular development of piglets up to an age of 84 d.

## Conclusion

In summary, the results of our study showed that 4 μg/kg of 1α,25(OH)2VD3-glycosides from *Solanum glaucophyllum* on top of 1000 IU VD can promoted the proliferation of testicular cells, the development of seminiferous tubules and had a positive effect on the weight and volume of early testicular development in piglets. Meanwhile, the proteomics method based on TMT markers was used to study the changes of protein composition in early testes of piglets with different dosages of 1α,25(OH)_2_VD_3_, provided as 1α,25(OH)_2_VD_3_-glycosides from plant origin. Bioinformatics tools were used to further characterize the differential abundance proteins. These DAPs may be potential proteomic markers that regulate early testicular development in piglets, such as CYP11A1, CYP19A1, CAT, PEX10, INSL3. Functional annotations indicated that identified DAPs were diverse, including the regulation of biological processes, redox processes, metabolic processes, and catalytic activity. These DAPs, as main intermediates or key enzymes, were involved in a variety of potential signalling pathways, including steroid hormone synthesis, steroid biosynthesis, peroxisome and fatty acid metabolism pathways. These pathways were closely related to testicular function, testicular development and spermatogenesis. Therefore, it can be speculated that the cumulative effect of 1α,25(OH)_2_VD_3_ in the testes plays an important regulatory role in early testicular development of piglets. The study also identified some candidate proteins that may be related to early testicular development regulation. These important candidate proteins could be further considered as the genetic biomarkers for the fecundity of boars.

## Supplementary Information

Supplementary Information 1.

Supplementary Information 2.

Supplementary Information 3.

Supplementary Information 4.

Supplementary Information 5.

Supplementary Information 6.

Supplementary Information 7.

Supplementary Information 8.

Supplementary Information 9.

Supplementary Information 10.

Supplementary Information 11.

Supplementary Information 12.

Supplementary Information 13.

## Data Availability

The datasets generated during and/or analysed during the current study are available from the corresponding author on reasonable request.

## Abbreviations

VD3: Vitamin D3
DAPs: Differentially abundant proteins
VDR: Vitamin D receptor
HE: Haematoxylin and eosin
AGC: Automatic gain control
GO: Gene Ontology
BP: Biological process
CC: Cellular component
MF: Molecular function
KEGG: Kyoto Encyclopaedia of Genes and Genomes
PPI: Protein–protein interactions
CYP11A1: The cholesterol side chain lyase
CYP19A1: Cytochrome P450 Family 19 Subfamily A Member 1
FDFT1: Farnesyl-Diphosphate Farnesyltransferase 1
CAT: Catalase
DHRS4: Dehydrogenase/Reductase 4
EHHADH: Enoyl-CoA Hydratase And 3-Hydroxyacyl CoA Dehydrogenase
HSD17B4: Hydroxysteroid 17-Beta Dehydrogenase 4
ECH1: Enoyl-CoA Hydratase 1
BCKDHA: Branched Chain Keto Acid Dehydrogenase E1 Subunit Alpha
CYP1A1: Cytochrome P450 Family 1 Subfamily A Member 1
INSL3: Insulin Like 3
PEX: Peroxisomes
CSN3: Casein Kappa
VLCFA: Very Long Chain Fatty Acids

## References

[1] Norman AW (2008). From vitamin D to hormone D: fundamentals of the vitamin D endocrine system essential for good health. Am. J. Clin. Nutr..

[2] Caprio M, Infante M, Calanchini M, Mammi C, Fabbri A (2017). Vitamin D: not just the bone. Evidence for beneficial pleiotropic extraskeletal effects. Eat Weight Disord..

[3] Anagnostis P, Karras S, Goulis DG (2013). Vitamin D in human reproduction: a narrative review. Int. J. Clin. Pract..

[4] Jensen MB (2014). Vitamin D and male reproduction. Nat. Rev. Endocrinol..

[5] Merke J, Hugel U, Ritz E (1985). Nuclear testicular 1,25-dihydroxyvitamin D3 receptors in Sertoli cells and seminiferous tubules of adult rodents. Biochem. Biophys. Res. Commun..

[6] Johnson JA, Grande JP, Roche PC, Kumar R (1996). Immunohistochemical detection and distribution of the 1,25-dihydroxyvitamin D3 receptor in rat reproductive tissues. Histochem. Cell Biol..

[7] Kidroni G, Har-Nir R, Menezel J, Frutkoff IW, Palti Z, Ron M (1983). Vitamin D3 metabolites in rat epididymis: high 24,25-dihydroxy vitamin D3 levels in the cauda region. Biochem. Biophys. Res. Commun..

[8] Oliveira AG, Dornas RA, Kalapothakis E, Hess RA, Mahecha GA, Oliveira CA (2008). Vitamin D3 and androgen receptors in testis and epididymal region of roosters (Gallus domesticus) as affected by epididymal lithiasis. Anim. Reprod. Sci..

[9] Hui J, Yang H, Guang J, Yanrong X, Xiaowei Q, Xiaolei Y, Wenbing Y (2015). The vitaminD receptor localization and mRNA expression in ram testis and epididymis. Anim. Reprod. Sci..

[10] Corbett ST, Hill O, Nangia AK (2006). Vitamin D receptor found in human sperm. Urology.

[11] Jensen MB, Nielsen JE, Jørgensen A, Meyts ERD, Kristensen DM, Jørgensen N, Skakkebaek NE, Juul A, Leffers H (2010). Vitamin D receptor and vitamin D metabolizing enzymes are expressed in the human male reproductive tract. Hum. Reprod..

[12] Christakos S, Dhawan P, Verstuyf A, Verlinden L, Carmeliet G (2016). Vitamin D: metabolism, molecular mechanism of action, and pleiotropic effects. Physiol. Rev..

[13] Boland R, Skliar M, Curino A, Milanesi L (2003). Vitamin D compounds in plants. Plant Sci..

[14] Gili V, Pardo VG, Ronda AC, Genaro PD, Bachmann H, Boland R, de Boland AR (2016). In vitro effects of 1α,25(OH)2VD3-glycosides from Solbone A (Solanum glaucophyllum leaves extract; Herbonis AG) compared to synthetic 1α,25(OH)2D3 on myogenesis. Steroids.

[15] Liu J, Like Qu, Meng L, Shou C (2019). Topoisomerase inhibitors promote cancer cell motility via ROS-mediated activation of JAK2-STAT1-CXCL1 pathway. J. Exp. Clin. Cancer Res..

[16] Naiwen Z, Ning J, Kai Z, Lili Z, Di Z, Xiaoyu S, Ying F, Ran C, Na Y, Xinyi W, Zhongyi C, Xun S, Zhaorong L, Qijun C (2020). Landscapes of protein posttranslational modifications of African trypanosoma parasites. iScience..

[17] Guanglei Z, Kebing Y, Zhaoshi J, Alicia C, Jenny Y, Connie H, Karen T, Robert S, Benjamin H, Elizabeth B, Deepak S, Carlos B, Jennie RL, Napoleone F (2013). Phosphoproteomic analysis implicates the mTORC2-FoxO1 axis in VEGF signaling and feedback activation of receptor tyrosine kinases. Sci. Sign..

[18] Yuexi W, Feng Y, Marina AG, Yingchun W, Therese C, Tao L, Yufeng S, Matthew EM, Daniel L-F, Theresa R, Ronald JM, Richard LK, David GC, Richard DS (2011). Reversed-phase chromatography with multiple fraction concatenation strategy for proteome profiling of human MCF10A cells. Proteomics.

[19] Andrew T, Jürgen S, Karsten K, Stefan K, Josef S, Günter S, Thomas N, Johnstone R, Karim A, Mohammed A, Christian H (2003). Tandem mass tags: a novel quantification strategy for comparative analysis of complex protein mixtures by MS/MS. Anal. Chem..

[20] Loïc D, Alexandre H, Virginie L, Natacha T, Karsten K, Denis FH, Pierre RB, Jean-Charles S (2008). Relative quantification of proteins in human cerebrospinal fluids by MS/MS using 6-plex isobaric tags. Anal. Chem..

[21] Tyanova S, Temu T, Cox J (2016). The MaxQuant computational platform for mass spectrometry-based shotgun proteomics. Nat. Protoc..

[22] Damian S, Annika LG, David L, Alexander J, Stefan W, Jaime H-C, Milan S, Nadezhda TD, John HM, Peer B, Lars JJ, von Christian M (2019). STRING v11: protein-protein association networks with increased coverage, supporting functional discovery in genome-wide experimental datasets. Nucleic Acids Res..

[23] Kanehisa M, Goto S (2000). KEGG: kyoto encyclopedia of genes and genomes. Nucleic Acids Res..

[24] Clark AM, Chuzel F, Sanchez P, Saez JM (1996). Regulation by gonadotropins of the messenger ribonucleic acid for P450 side-chain cleavage, P450(17) alpha-hydroxylase/C17, 20-lyase, and 3 beta-hydroxysteroid dehydrogenase in cultured pig Leydig cells. Biol. Reprod..

[25] Yiqing P, Chen X (2005). Role of estrogen in male reproduction. Natl. J. Androl..

[26] Carreau S, Wolczynski S, Galeraud-Denis I (2010). Aromatase, oestrogens and human male reproduction. Philos. Trans. R. Soc. Lond. B Biol. Sci..

[27] Xiuli G, Honggang L, Xi C, Xue Z, Fen M, Mingzhu J, Chengliang X (2019). PEX10, SIRPA-SIRPG, and SOX5 gene polymorphisms are strongly associated with nonobstructive azoospermia susceptibility. J. Assist. Reprod. Genet..

[28] Samanta L, Roy A, Chainy GB (1999). Changes in rat testicular antioxidant defence profile as a function of age and its impairment by hexachlorocyclohexane during critical stages of maturation. Andrologia.

[29] Dumont L, Oblette A, Rondanino C, Jumeau F, Bironneau A, Liot D, Duchesne V, Wils J, Rives N (2016). Vitamin A prevents round spermatid nuclear damage and promotes the production of motile sperm during in vitro maturation of vitrified pre-pubertal mouse testicular tissue. Mol. Hum. Reprod..

[30] França LR, Silva-Jr VA, Chiarini-Garcia H, Garcia SK, Debeljuk L (2000). Cell proliferation and hormonal changes during postnatal development of the testis in the pig. Biol. Reprod..

[31] Yan S, Xianzhong W, Jianyun W, Pei Z, Yanhua S, Jiahua Z (2005). Expression of glial cell line-derived neurotrophic factor(GDNF) in boar testes during different stages. J. Agric. Biotech..

[32] Xiaojuan M, Lindahl M, Hyvönen ME, Parvinen M, de Rooij DG, Hess MW, Raatikainen-Ahokas A, Sainio K, Rauvala H, Lakso M, Pichel JG, Westphal H, Saarma M, Sariola H (2000). Regulation of cell fate decision of undifferentiated spermatogonia by GDNF. Science.

[33] Weiwei S, Lulu C, Wei Z, Rong W, Goltzman D, Dengshun M (2015). Active vitamin D deficiency mediated by extracellular calcium and phosphorus results in male infertility in young mice. Am. J. Physiol. Endocrinol. Metab..

[34] Sakamoto H, Ogawa Y, Yoshida H (2008). Relationship between testicular volume and testicular function: comparison of the Prader orchidometric and ultrasonographic measurements in patients with infertility. Asian J. Androl..

[35] Kerketta S, Singh M, Patel BHM, Dutt T, Upadhyay D, Bharti PK, Sahu S, Kamal R (2015). Relationships between age, body measurements, testicular measurements and total ejaculation of semen in local goat of Rohilkhand region. Small Rumin. Res..

[36] Coe PH (1999). Associations among age, scrotal circumference, and proportion of morphologically normal spermatozoa in young beef bulls during an initial breeding soundness examination. J. Am. Vet. Med. Assoc..

[37] França LR, Avelar GF, Almeida FFL (2005). Spermatogenesis and sperm transit through the epididymis in mammals with emphasis on pigs. Theriogenology.

[38] Qiuping M, Jisheng X, Ruiya H, Weixiang S, Jingai Y (2007). Research progress and future application of Sertoli cells. CRTER.

[39] Walker WH (2011). Testosterone signaling and the regulation of spermatogenesis. Spermatogenesis..

[40] Zengming Y, Qingyuan S, Guoliang X (2005). Reproductive Biology.

[41] Strushkevich N, MacKenzie F, Cherkesova T, Grabovec I, Usanov S, Park HW (2011). Structural basis for pregnenolone biosynthesis by the mitochondrial monooxygenase system. Proc. Natl. Acad. Sci. U S A..

[42] Miller WL (1988). Molecular biology of steroid hormone synthesis. Endocr. Rev..

[43] Black SM, Harikrishna JA, Szklarz GD, Miller WL (1994). The mitochondrial environment is required for activity of the cholesterol side-chain cleavage enzyme, cytochrome P450scc. Proc. Natl. Acad. Sci. U S A..

[44] Farkash Y, Timberg R, Orly J (1986). Preparation of antiserum to rat cytochrome P-450 cholesterol side chain cleavage, and its use for ultrastructural localization of the immunoreactive enzyme by protein A-gold technique. Endocrinology.

[45] Baravalle R, Di Nardo G, Bandino A, Barone I, Catalano S, Andò S, Gilardi G (2017). Impact of R264C and R264H polymorphisms in human aromatase function. J. Steroid. Biochem. Mol. Biol..

[46] Kinuta K, Tanaka H, Moriwake T, Aya K, Kato S, Seino Y (2000). Vitamin D is an important factor in estrogen biosynthesis of both female and male gonads. Endocrinology.

[47] Zanatta L, Bouraïma-Lelong H, Delalande C, Silva FRMB, Carreau S (2011). Regulation of aromatase expression by 1α,25(OH)2Vitamin D3 in rat testicular cells. Reprod. Fertil. Dev..

[48] Hess RA, Bunick D, Lee KH, Bahr J, Taylor JA, Korach KS, Lubahn DB (1997). A role for oestrogens in the male reproductive system. Nature.

[49] Sharpe RM (1998). The roles of oestrogen in the male. Trends Endocrinol. Metab..

[50] Hofer D, Münzker J, Schwetz V, Ulbing M, Hutz K, Stiegler Ph, Zigeuner R, Pieber TR, Müller H, Obermayer-Pietsch B (2014). Testicular synthesis and vitamin D action. J. Clin. Endocrinol. Metab..

[51] Brooks K, Burns G, Spencer TE (2015). Biological roles of hydroxysteroid (11-Beta) dehydrogenase 1 (HSD11B1), HSD11B2, and glucocorticoid receptor (NR3C1) in sheep conceptus elongation. Biol. Reprod..

[52] Wanders RJA, Waterham HR (2006). Biochemistry of mammalian peroxisomes revisited. Annu. Rev. Biochem..

[53] Titorenko VI, Rachubinski RA (2001). The life cycle of the peroxisome. Nat. Rev. Mol. Cell Biol..

[54] Eckert JH, Erdmann R (2003). Peroxisome biogenesis. Rev. Physiol. Biochem. Pharmacol..

[55] Haiyang C, Zhonghua L, Xun H (2010). Drosophila models of peroxisomal biogenesis disorder: peroxins are required for spermatogenesis and very-long-chain fatty acid metabolism. Hum. Mol. Genet..

[56] Shimozawa N, Imamura A, Zhang Z, Suzuki Y, Orii T, Tsukamoto T, Osumi T, Fujiki Y, Wanders RJ, Besley G, Kondo N (1999). Defective PEX gene products correlate with the protein import, biochemical abnormalities, and phenotypic heterogeneity in peroxisome biogenesis disorders. J. Med. Genet..

[57] Sargent G, Zutphen TV, Shatseva T, Zhang L, Giovanni VD, Bandsma R, Kim PK (2016). PEX2 is the E3 ubiquitin ligase required for pexophagy during starvation. J. Cell Biol..

[58] Hanson MG, Fregoso VL, Vrana JD, Tucker CL, Niswander LA (2014). Peripheral nervous system defects in a mouse model for peroxisomal biogenesis disorders. Dev. Biol..

[59] Poulos A (1995). Very long chain fatty acids in higher animals–a review. Lipids.

[60] Steven H, Henning S, Karel DG, Guido V, Florian G, Paul PVV, Myriam B (2006). Peroxisomal multifunctional protein 2 is essential for lipid homeostasis in Sertoli cells and male fertility in mice. Endocrinology.

[61] Datar J, Regassa A, Kim WK, Taylor CG, Zahradka P, Suh M (2017). Lipid metabolism is closely associated with normal testicular growth based on global transcriptome profiles in normal and underdeveloped testis of obese zucker (fa/fa) rats. Lipids.

[62] Borges CC, Salles AF, Bringhenti I, Mandarim-DE-Lacerda CA, Aguila MB (2018). Vitamin D deficiency increases lipogenesis and reduces beta-oxidation in the liver of diet-induced obese mice. J. Nutr. Sci. Vitaminol (Tokyo)..

[63] Chihara M, Otsuka S, Ichii O, Kon Y (2013). Vitamin A deprivation affects the progression of the spermatogenic wave and initial formation of the blood-testis barrier, resulting in irreversible testicular degeneration in mice. J. Reprod. Dev..

[64] Livera G, Rouiller-Fabre V, Pairault C, Levacher C, Habert R (2002). Regulation and perturbation of testicular functions by vitamin A. Reproduction.

[65] Pathirana IN, Kawate N, Büllesbach EE, Takahashi M, Hatoya S, Inaba T, Tamada H (2012). Insulin-like peptide 3 stimulates testosterone secretion in mouse Leydig cells via cAMP pathway. Regul. Pept..

