# Supplementary material for: Proteomics analysis reveals the effect of 1α,25(OH)2VD3-glycosides on development of early testes in piglets
Source: Sci Rep. 2021 May 31;11:11341. doi: 10.1038/s41598-021-90676-8 (PMC8167176; doi:10.1038/s41598-021-90676-8)
Supplement: Supplementary file 10 — Supplementary Information 10. [file 41598_2021_90676_MOESM10_ESM.html]

forceNetwork
