# Supplementary material for: Proteomics analysis reveals the effect of 1α,25(OH)2VD3-glycosides on development of early testes in piglets
Source: Sci Rep. 2021 May 31;11:11341. doi: 10.1038/s41598-021-90676-8 (PMC8167176; doi:10.1038/s41598-021-90676-8)
Supplement: Supplementary file 13 — Supplementary Information 13. [file 41598_2021_90676_MOESM13_ESM.pdf]

Supporting information

Supporting tables：

Table S1. All MS identified information.

Table S2. All identified differentially abundant proteins statistics information.

Table S3. All protein GO terms_Level2 classify.

Table S4. Protein GO terms_Level2 classify of S1 vs S2.

Table S5. Protein GO terms_Level2 classify of S1 vs S3.

Table S6. Protein GO terms_Level2 classify of S1 vs S4.

Table S7. DAPs of KEGG pathway enrichment between S1 vs S2.

Table S8. DAPs of KEGG pathway enrichment between S1 vs S3.

Table S9. DAPs of KEGG pathway enrichment between S1 vs S4.

Supporting figures：

Figure S1. The figures of raw western blots(unprocessed versions).

Figure S1. The figures of raw western blots(red boxes labelled).

Figure S1. The figures of raw western blots(labelled).

Supporting material：

N1. PPI network of S1 vs S2.

N2. PPI network of S1 vs S3.

N3. PPI network of S1 vs S4.

In addition, We had uploaded all the original proteomics data and peptides information to the PRIDE public database, and reviewers can log in and view with the following account and password:

Account: whliu@mail.hzau.edu.cn
Password: hnyx111476

Figure S1.The figures of raw western blots(unprocessed versions)

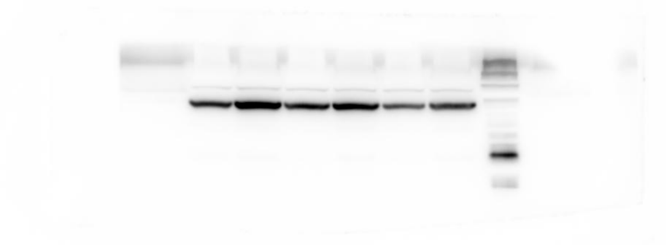

CAT

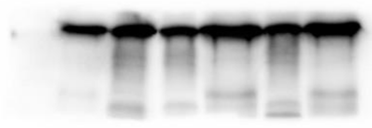

DHRS4

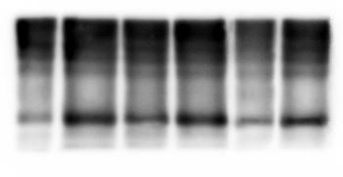

INSL3

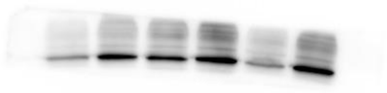

HSD17B4

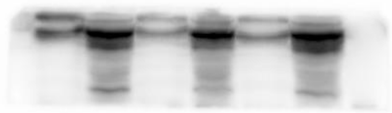

CYP19A1

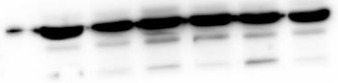

β-Actin1

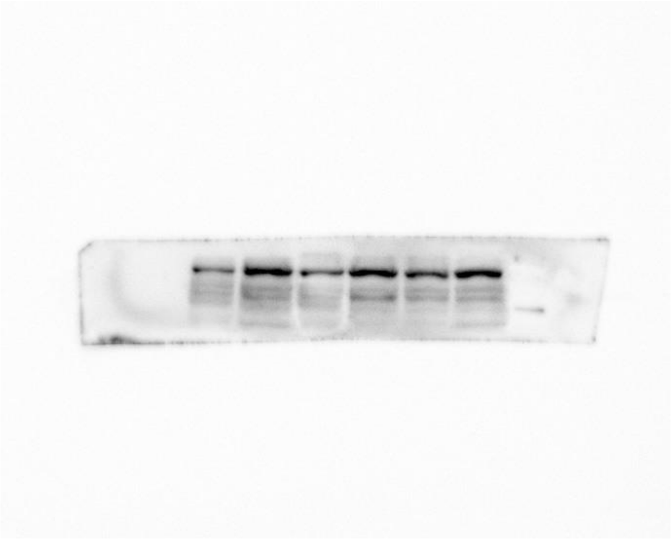

FDFT1

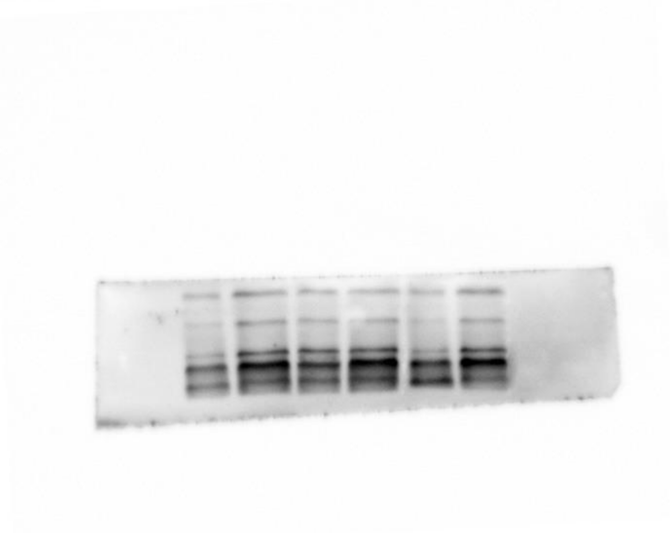

PEX10

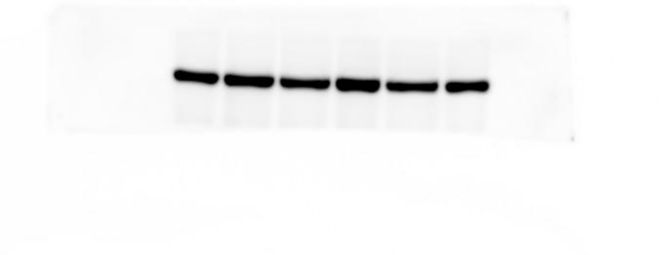

β-Actin2

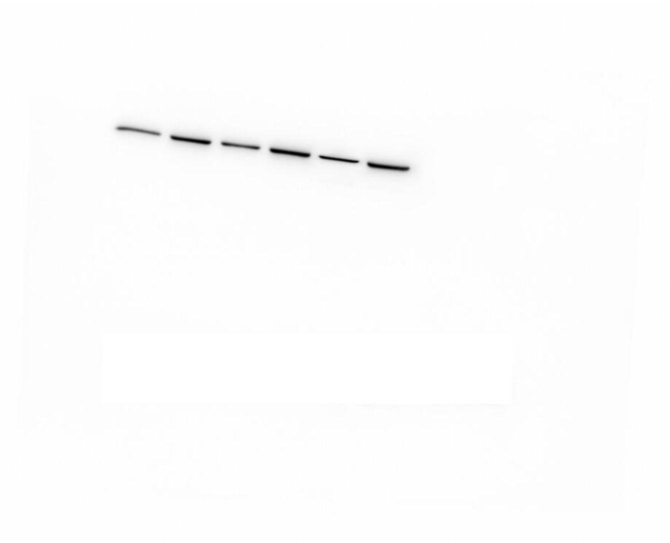

CYP11A1

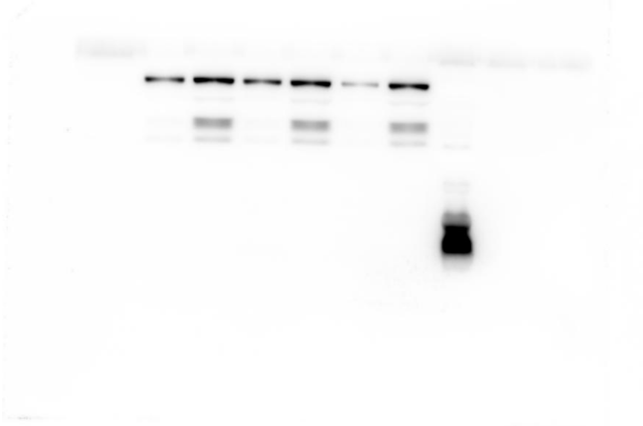

PHYH

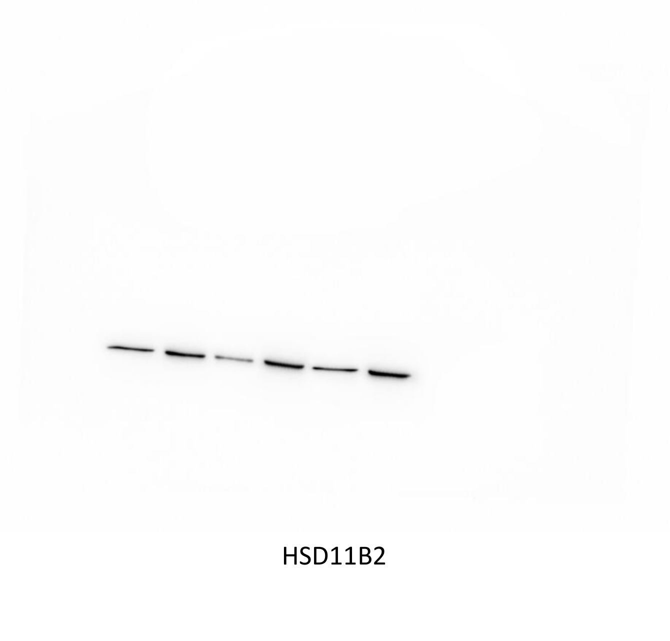

HSD11B2

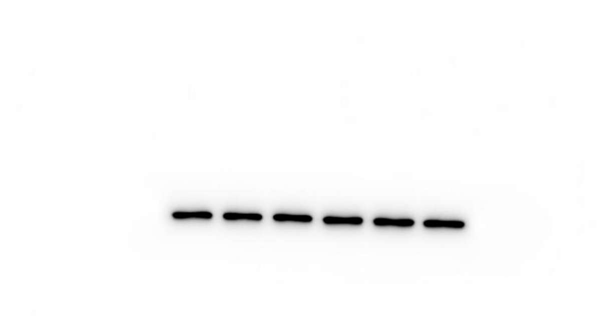

β-Actin3

Figure S1.The figures of raw western blots(red boxes labelled)

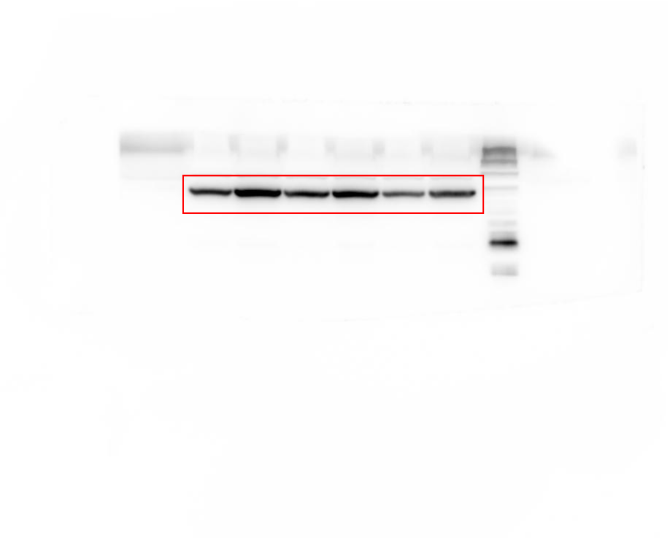

CAT

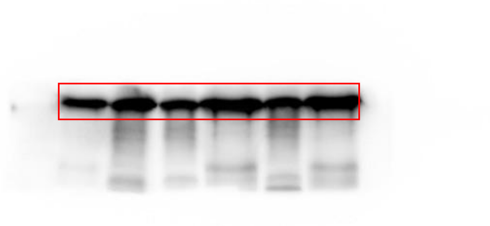

DHRS4

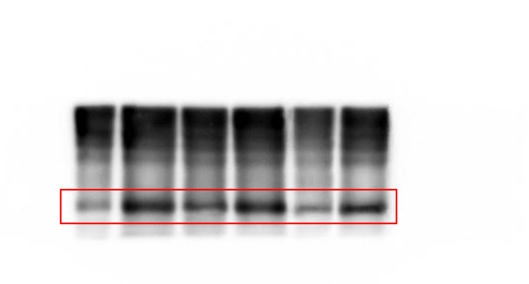

INSL3

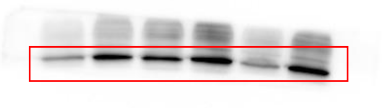

HSD17B4

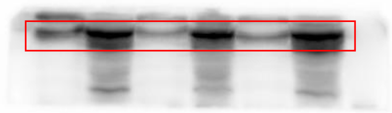

CYP19A1

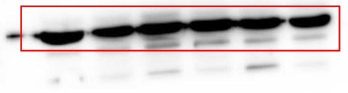

β-Actin1

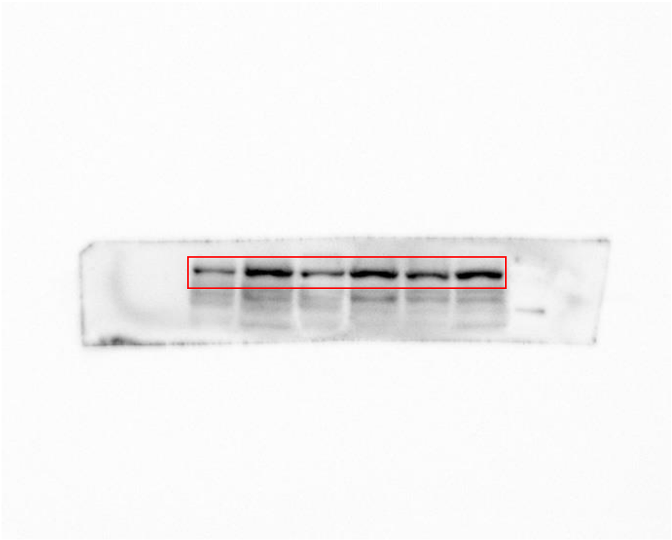

FDFT1

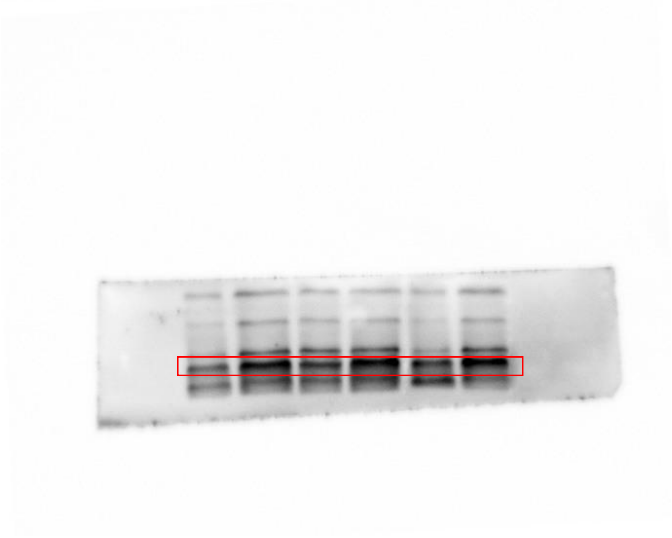

PEX10

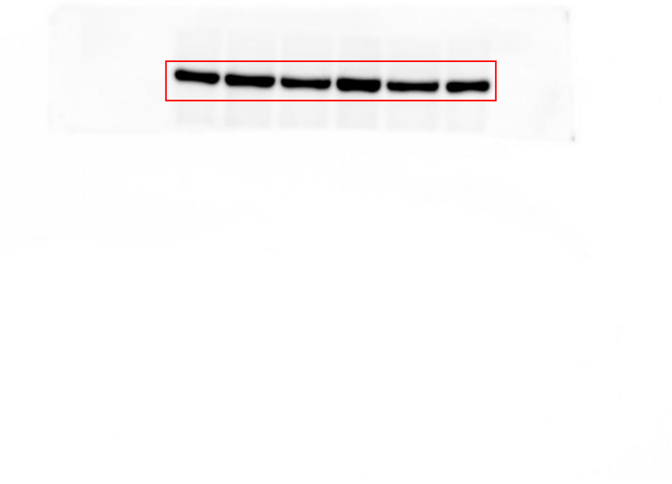

β-Actin2

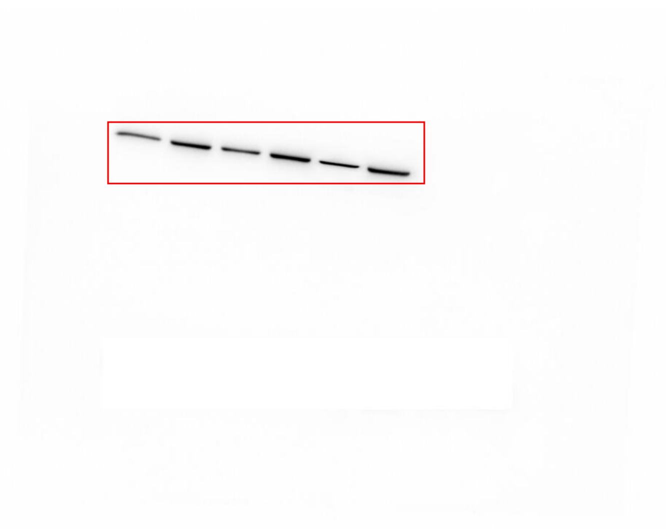

CYP11A1

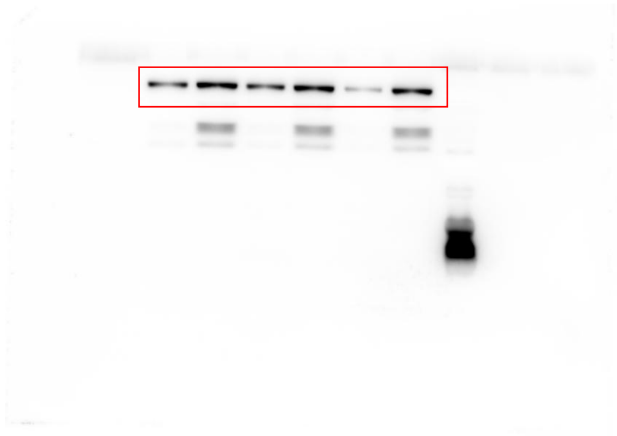

PHYH

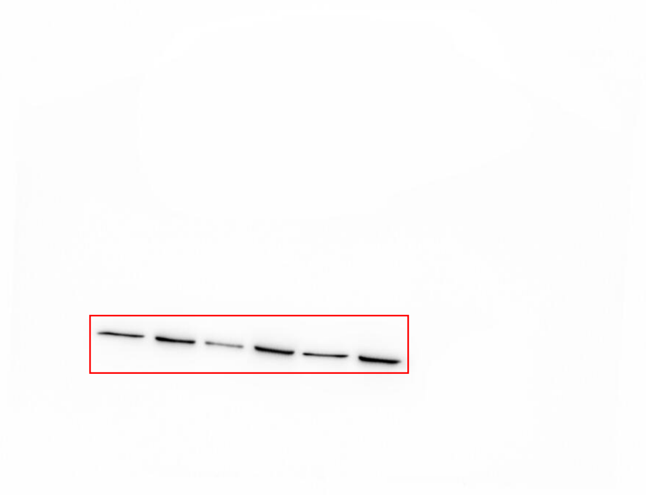

HSD11B2

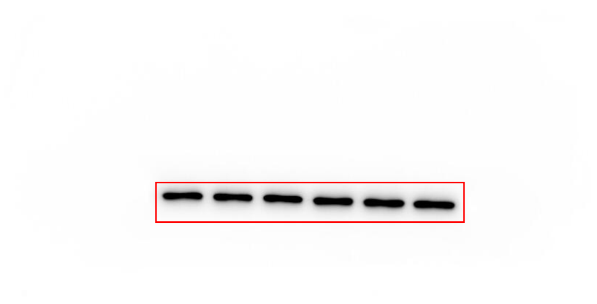

β-Actin3

Figure S1.The figures of raw western blots(labelled)

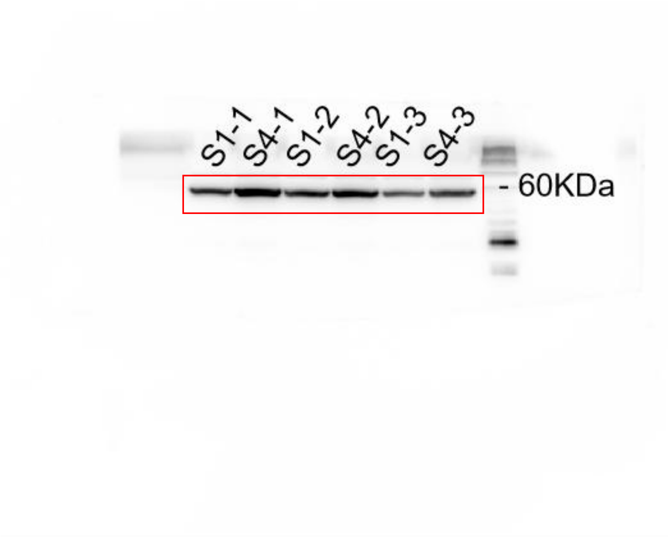

CAT

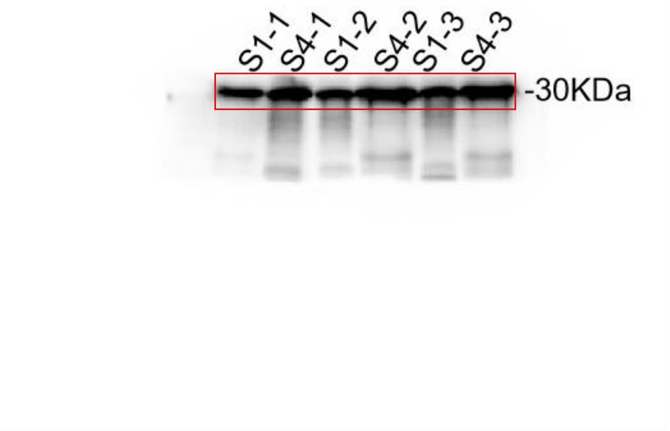

DHRS4

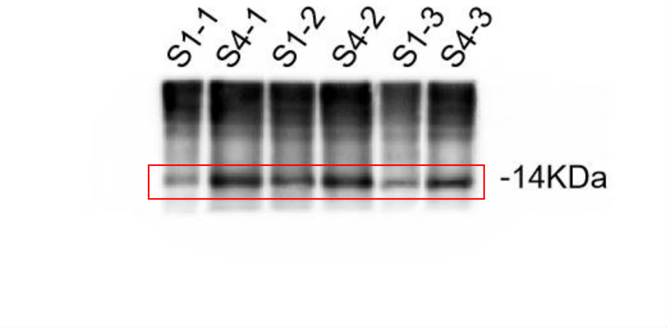

INSL3

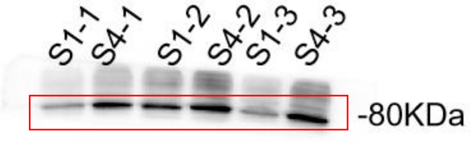

HSD17B4

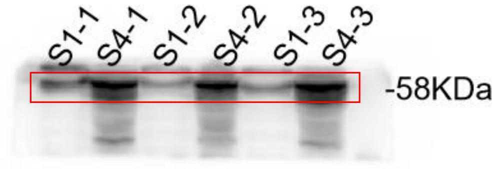

CYP19A1

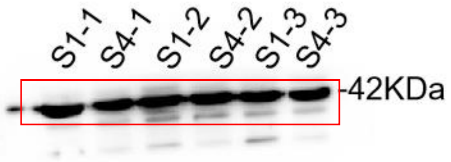

β-Actin1

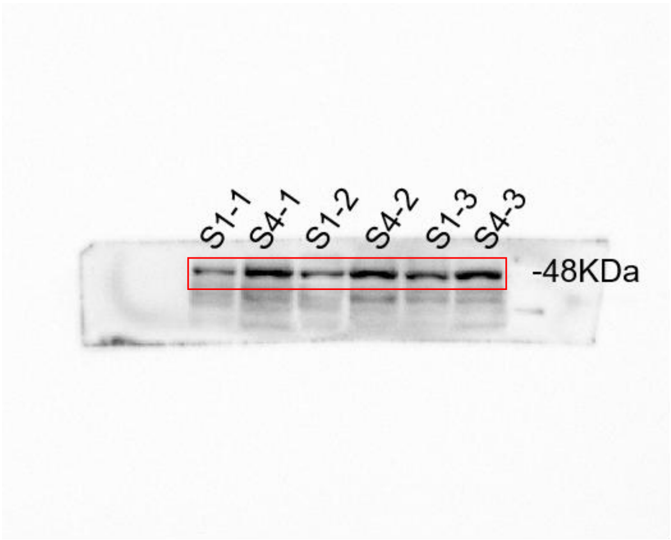

FDFT1

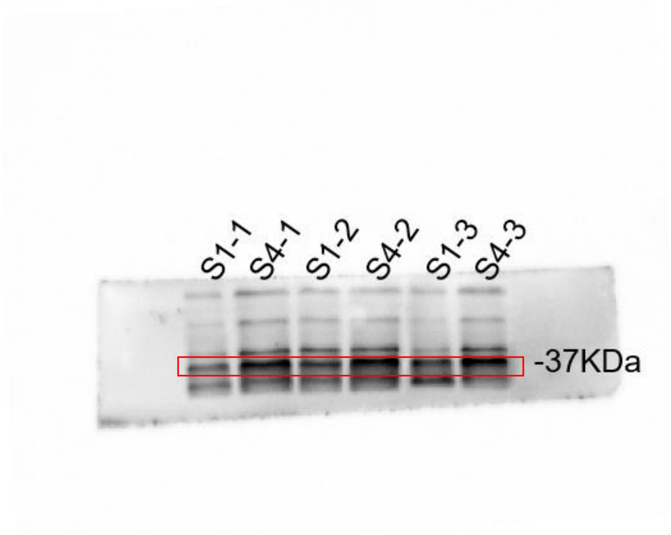

PEX10

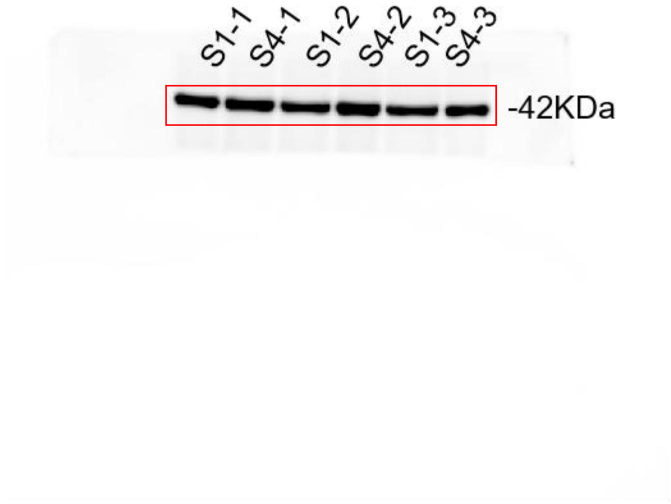

β-Actin2

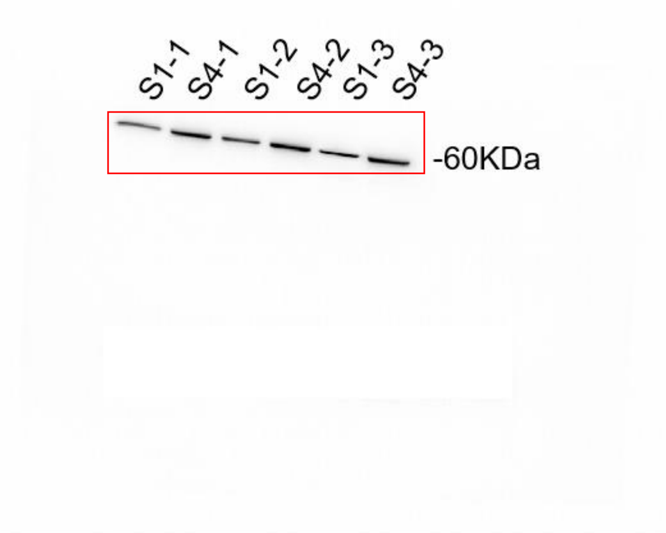

CYP11A1

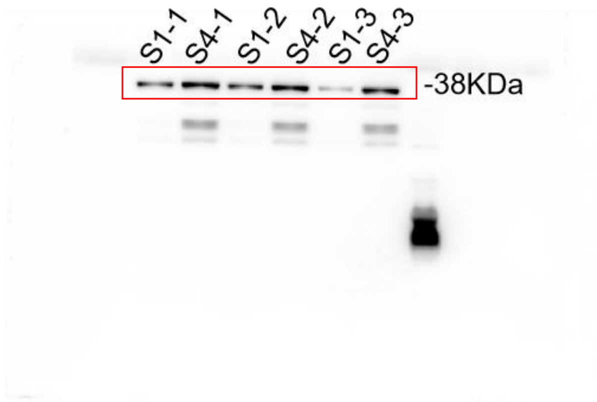

PHYH

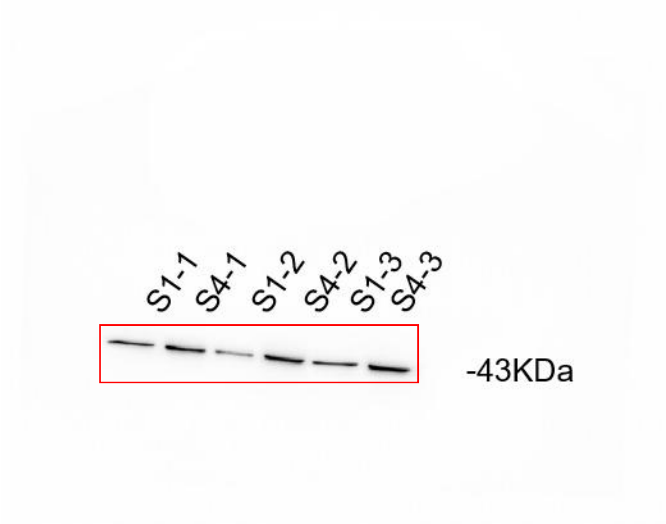

HSD11B2

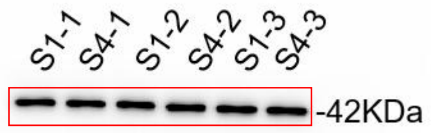

β-Actin3
